# Suicide Assessment and Management Team-Based Learning Module

**DOI:** 10.15766/mep_2374-8265.10952

**Published:** 2020-08-20

**Authors:** Sarah Lerchenfeldt, Suzan Kamel-ElSayed, Gustavo Patino, David M. Thomas, Jolyn Wagner

**Affiliations:** 1 Assistant Professor, Department of Foundational Medical Studies, Oakland University William Beaumont School of Medicine; 2 Associate Professor, Department of Foundational Medical Studies, Oakland University William Beaumont School of Medicine; 3 Assistant Professor, Department of Foundational Medical Studies, Oakland University William Beaumont School of Medicine; Assistant Professor, Department of Neurology, Oakland University William Beaumont School of Medicine; 4 Interim Associate Dean for Preclinical Medical Education, Office of Medical Education, Oakland University William Beaumont School of Medicine; 5 Assistant Professor, Department of Psychiatry, Oakland University William Beaumont School of Medicine

**Keywords:** Suicide, Suicide Risk Assessment, Suicide Management, Suicide Treatment, Team-Based Learning, Clinical Reasoning/Diagnostic Reasoning, Communication Skills, Ethics, Evidence-Based Medicine, Patient Safety

## Abstract

**Introduction:**

Suicide is a global health problem that health care providers must feel comfortable addressing. Unfortunately, many health care providers are not equipped to assess and treat patients at risk for suicide due to lack of training and education. Interactive resources are needed to educate health professions students about the management of suicidal patients.

**Methods:**

The suicide assessment and management team-based learning (TBL) module was developed to address the gap in suicide education. After completing the module, students were able to identify key elements for a comprehensive assessment of a patient's risk for suicide and to discuss clinical management for a suicidal patient. The activity was designed for second-year medical students during a psychopathology course, the last organ-system course prior to clerkships. This module could also be used or modified to meet the educational requirements for other health professions, including medical residents, nurse practitioner students, and physician assistant students.

**Results:**

A total of 342 students among 62 teams participated in the TBL over a period of 3 consecutive years. The class averages for the individual Readiness Assurance Test ranged from 80% to 88%. The class averages for the team Readiness Assurance Test and application questions were comparable across all 3 years. Course evaluations showed the TBL helped students think critically and integrate information to prepare them for their future careers.

**Discussion:**

Overall, this TBL was an effective educational tool that stimulated high-quality discussion, in which students remained engaged and asked thought-provoking questions.

## Educational Objectives

By the end of this activity, learners will be able to:

1. Identify risk factors associated with suicide.
2. Compare and contrast subgroups of the population that are at increased risk for suicide, including children, adolescents, and elderly individuals, and discuss how to eliminate disparities among these vulnerable groups.
3. Indicate which medications are used to reduce the risk of suicide and which are used in the clinical management of a suicidal individual.
4. Discuss the key elements that must be evaluated in order to conduct a comprehensive assessment of a patient's risk for suicide.
5. Compare the major treatment strategies (acute and long-term) utilized to optimally treat patients who are assessed and believed to be at significant risk for suicide.
6. Create a clinical management plan for a suicidal patient using evidence-based medicine.
7. Discuss differences in state policies for efficient and effective continuity of care.
8. Participate in the team-based learning activity in a professional and respectful manner.
9. Engage the material by critically evaluating its content and employing peer teaching throughout the session.

## Introduction

According to the Centers for Disease Control and Prevention, suicide is a leading cause of death in the United States; the rate of suicide has increased by more than 30% in half of all states since 1999.^1^ For this reason, it is extremely important that health care professionals are properly educated on how to manage patients at risk for suicide. While psychiatrists may play a large role in suicide assessment and management (SAM), many other health care professionals, including medical professionals in the emergency department or primary care setting, will also encounter patients at risk for suicide.^2,3^ Many patients follow up with their primary care provider prior to attempting or dying by suicide.^4–9^ It is essential for health care professionals to possess the knowledge and skills to manage suicidal patients; unfortunately, training in this area is often limited, and health care professionals may not be well equipped to properly handle such patients.^4^

The current literature describing the availability and success of suicide prevention education for undergraduate medical students is underwhelming.^4^ According to Gramaglia and Zeppegno, there are gaps in training students about suicide prevention and management.^2^ In order to address these gaps, it is thought that interactive learning techniques can better educate students about the difficult and multifaceted world of suicide.^2^ There are very few such resources available in *MedEdPORTAL*. One available resource, developed by Owen, Pheister, and Simons, covers a multidisciplinary approach to risk assessment and the emotional aftermath of patient suicide.^10^ This resource includes a PowerPoint lecture, a case-based small-group discussion, and role-play used throughout a 4-hour symposium. There is also a team-based learning (TBL) module available in *MedEdPORTAL* that focuses on suicide risk assessment but does not address the clinical management and major treatment strategies for a suicidal patient.^11^ Another *MedEdPORTAL* resource concentrates on physician wellness to address physician burnout and suicide but is not designed to train students how to identify and manage suicidal patients.^12^ Additionally, studies have shown that simulation and standardized patients may be effective strategies to educate students about assessing and managing patients at risk for suicide, although the resources themselves are not publicly available for use.^2,13,14^ Overall, it is clear that additional interactive resources are needed to educate health professional students about the management of suicidal patients.

TBL can be a very effective and interactive instructional strategy to educate students about suicide assessment, prevention, and management. Going beyond simply covering content, TBL instead focuses on providing students the opportunity to practice solving complex problems.^15^ TBL is an interactive instructional strategy that engages students with lifelike scenarios they will encounter in their chosen profession.^16^ Aside from increased student engagement and interaction, there are several other advantages to using TBL. For example, TBL has been shown to improve communication among students and increase scores on licensing examinations; in addition, it allows for increased student interaction without requiring a large number of participating faculty.^17–20^

This TBL module on SAM not only offered an interactive learning strategy for students but also addressed an extremely important topic that may not be sufficiently covered in health profession education. It covered both the key elements that must be evaluated in order to conduct a comprehensive assessment of a patient's risk for suicide and also the clinical management of a suicidal patient, including acute and long-term treatment strategies. Students were expected to engage with the material by critically evaluating its content and employing peer teaching throughout the session. Although the module was designed for second-year medical students during a psychopathology course, it could be used or modified to meet the educational requirements for other health professions as well, including, but not limited to, nurse practitioner and physician assistant students.

## Methods

### Curricular Context

At Oakland University William Beaumont School of Medicine (OUWB), organ-system courses were used during the preclinical curriculum, which vertically integrated the basic and clinical sciences. The curriculum map of the organ-system courses for the first 2 years is below.

• First year of medical school (in order of occurrence):
  ○ Anatomical foundations of clinical practice.
  ○ Biomedical foundations of clinical practice.
  ○ Hematopoietic and lymphoid.
  ○ Neuroscience 1.
  ○ Cardiovascular.
  ○ Respiratory.
• Second year of medical school (in order of occurrence):
  ○ Renal and urinary.
  ○ Gastroenterology and hepatology.
  ○ Endocrinology.
  ○ Male and female reproductive.
  ○ Neuroscience 2.
  ○ Musculoskeletal, connective tissue, and skin.
  ○ Behavioral sciences.
  ○ Psychopathology.

The psychopathology course presented the central concepts of contemporary mental health diagnosis as outlined in the fifth edition of the *Diagnostic and Statistical Manual of Mental Disorders,* in which emphasis was placed on descriptive psychopathology, including etiology, phenomenology, symptom profiles, and course of disease.^21^ Below is a list of the sessions delivered throughout the course to provide further understanding on the content covered:

• Mental status exam.
• Mood disorders.
• Antidepressant pharmacology.
• Somatic symptom disorders.
• Personality disorders.
• Psychotic disorders.
• Antipsychotics pharmacology.
• Anxiety disorders.
• Eating disorders.
• Child and adolescent psychiatry.
• Substance abuse disorders.
• Drugs of abuse pharmacology.
• Drugs of abuse patient panel.
• Drugs of abuse TBL.
• SAM TBL.

The SAM TBL module was required for second-year medical students enrolled in the 2-week psychopathology course, offered near the end of their second semester.

The TBL was developed by two OUWB faculty members with different areas of expertise: psychiatry and pharmacology. Both were course directors at the time the module was created. In addition, members of the TBL oversight team assisted with the improvement and facilitation of the TBL. All members of the TBL oversight team were certified Team-Based Learning Collaborative (TBLC) trainer-consultants, having completed a rigorous development program to mentor and support others in implementing TBL. The course directors and TBL facilitators felt strongly that the TBL should be placed at the end of the course, to ensure students had a comprehensive and thorough understanding of mental health diagnosis. Each year, the TBL was evaluated based on exam statistics, quality of discussion, and student feedback.

By the end of the SAM TBL module, students were able to identify key elements for a comprehensive assessment of a patient's risk for suicide and to discuss the clinical management and available treatment strategies for a suicidal patient.

### Team Formation

The TBLC had three recommendations for creating teams, all of which OUWB closely followed for optimal group formation. First, the TBLC recommended that all assets and liabilities be divided across each team. To avoid preexisting, cohesive subgroups, the TBLC also recommended that teams not be self-selected by students. Lastly, in an effort to ensure students understood that no teams were given special advantages, it was recommended that students be made aware of the selection process.^22^ To make sure these recommendations were followed, GRumbler, a free computer software program developed by Malcolm Sparrow, was used to create well-balanced teams based on academic experience, health care experience, and gender.^23^ The students were notified that GRumbler was used for team formation. As recommended by the TBLC, to maximize individual resources while maintaining individual participation, each team consisted of five to seven students.^22^ Teams remained together for an entire academic year, since, as Levine noted, that it was advantageous for groups to stay together for extended periods of time in order to benefit from the strengths of each teammate.^24^

### Description of Advance Preparation Resources

The session objectives and preparatory assignments for the SAM TBL were provided to the students at least 1 week prior to the scheduled TBL activity (Appendix A). For the required preparatory reading, students were expected to read sections from the second edition of *The American Psychiatric Publishing Textbook of Suicide Assessment and Management.*^25^ All students had access to the required textbook through the school's medical library website.

Below is a more detailed description of the advance preparatory material:

• Required textbook: The American Psychiatric Publishing Textbook of Suicide Assessment and Management.^25^
  ○ Chapter 1: Suicide Risk Assessment: Gateway to Treatment and Management, pp. 12–21 (Systematic Suicide Risk Assessment, Populations at Risk for Suicide).^26^
  ○ Chapter 12: Psychopharmacotherapy and Electroconvulsive Therapy, pp. 216–218, 222–226 (Pharmacological Treatment, Electroconvulsive Therapy).^27^
  ○ Chapter 20: Children, Adolescents, and College Students, p. 350 (Table 20.1).^28^
  ○ Chapter 21: The Elderly, pp. 378–379 (Management of Suicide Risk in Late Life—Assessment and Intervention, Prevention of Suicide in Late Life).^29^

### Description of Readiness Assurance Process and Immediate Feedback

The Readiness Assurance Test (RAT) questions were designed to ensure that students read the assigned chapters and were prepared to work through the patient cases. The questions were written to assess the students' understanding of important concepts, such as suicide risk assessment, including risk factors for more vulnerable populations. While the questions were not difficult in nature, they helped confirm that students had a fundamental understanding of important factors to consider when evaluating and designing a treatment plan for an individual at risk for suicide.

Students were given the individual RAT (iRAT) at the beginning of the TBL activity.^30^ The iRAT for the SAM TBL contained a total of five multiple-choice questions. Each question was written to assess the students' understanding of the required portion of the advance preparation assignment (Appendix B). The iRAT followed the recommended process for testing conditions; therefore, Scantron forms or another approved testing software could be used. After the iRAT was complete, the team RAT (tRAT) was administered. With the tRAT, students took the same multiple-choice test with their previously assigned teams.^25^ Similar to a scratch-off lottery ticket, the tRAT used scratch-and-win type answer cards known as Immediate Feedback Assessment Technique (IF-AT) cards.^31^ The IF-AT cards were meant to provide immediate feedback about the correct answer to each question. Teams continued answering each question until a star was revealed, indicating it was the correct choice. If the correct answer was revealed on the first attempt, the team was awarded full credit; otherwise, the team progressively lost credit with each additional attempt to find the correct answer. Both the iRAT and the tRAT were closed book since they were part of the students' course grade; therefore, students were not allowed to use any notes, books, or other materials.

After the tRAT was complete, students were given the opportunity to ask facilitators about any confusing concepts that were encountered. The IF-AT cards made it possible for instructors to quickly identify areas of difficulty by analyzing how many attempts it took for each team to determine the correct answer. It was imperative that students have a clear understanding of the preparatory assignment prior to the application portion of the TBL activity, as those problems were more complex in nature.

In some cases, teams felt RAT questions were not appropriate, whether due to unclear wording, a disagreement with the correct answer, or other reasons. These teams filed an appeal within 24 hours after the TBL activity was completed (Appendix C). The appeal could not be filed during the TBL session as the teams were expected to concentrate on the application phase of the TBL. The facilitators were expected to review and respond to the appeal in a timely manner. Individual students were not allowed to file an appeal.

### Description of Team Application Activities

The application phase of the TBL was where students spent the majority of their time. In this phase, the application exercises required students to use their foundational knowledge, gained from the preparatory reading and RAT, to solve complex problems.^30^ The application phase was an extremely important part of the TBL activity, as it promoted the development of critical thinking and problem-solving skills. The application questions adhered to very specific criteria known as the 4 S's.^30^ The 4 S's stand for significant problem, same problem, specific choice, and simultaneous report. A more detailed description of the 4 S's is provided below:

1. Significant problem:
  • Questions must cover important concepts that were considered relevant to the students.
2. Same problem:
  • All students worked on the same question at the same time.
  • Created an enthusiastic environment.
  • Allowed for meaningful discussion and/or debate.
3. Specific choice:
  • Required students to use and improve their critical thinking and problem-solving skills.
4. Simultaneous report:
  • Prevented teams from being influenced by another team's decision.
  • Prevented teams from changing their answers.

In the SAM TBL, the application questions (Appendix D) were multiple choice; therefore, the teams reported their decision by holding up colored letter cards indicating their specific choice. The facilitators were expected to ask teams to discuss their answer choice with the rest of the class. Different teams were called on throughout the TBL, although teams were also allowed to volunteer to discuss their answers to further promote discussion. In some cases, more than one answer was considered correct (to some degree) for the application exercises; therefore, we found that ungraded application exercise questions promoted better discussion while decreasing some of the anxiety associated with an assessed activity.

### Facilitation Schema

• Phase 1:
  ○ Advance preparatory assignment: Objectives and advance preparatory material were assigned at least 1 week prior to the scheduled TBL module.
• Phase 2:
  ○ iRAT: Students were given 75 seconds per question.
  ○ tRAT: Students were given the amount of time it took to complete the iRAT plus 1 additional minute. Based on experience, we found that 1 additional minute allowed enough time for an effective team discussion.
  ○ Clarification of difficult concepts: Lasted a total of 5–10 minutes.
• Phase 3:
  ○ Application exercise: The remainder of the class period.
• Postsession:
  ○ Appeals: Students were given 24 hours to complete question appeals. Facilitators responded to appeals in a timely manner.

In order to continuously improve the TBL, several methods were used to evaluate the effectiveness of the module. Statistics from exam score reports for the iRAT/tRAT and the final exam (i.e., retired test questions from the National Board of Medical Examiners) were used each year to assess student learning. The quality of student learning was also assessed based on observation of student interaction and class discussion during the application exercises. In addition, data-analysis reports for the course and faculty providing anonymous student comments were used. Based on this information, minor changes were made to the TBL annually.

## Results

Overall, a total of 342 students and 62 teams participated in the SAM TBL over a period of 3 consecutive years. The class averages for the TBL were similar each year. In 2016, the averages were 83% and 99% for the iRAT and tRAT, respectively. In 2017, the average for the iRAT was 80%, and the average for the tRAT was 99%. Similarly, in 2018, the averages for the iRAT and tRAT were 88% and 98%, respectively. While the application exercises were not graded activities, the response rates for each question were comparable across all 3 years. In general, there was a good mix of answers for the application questions, which promoted great discussion. In most cases, the majority of teams chose the correct answers for the application questions, although a few questions could be simplified as most teams chose one of the distractors. Since the application questions were not graded, there was less student stress; therefore, there were many meaningful discussions throughout the entire TBL despite several teams choosing incorrect answers for certain questions. More information about the exam statistics for the iRAT and tRAT and response rates for the application exercises are in Appendices E and F. Explanations for the answers to the application questions are in Appendix G.

Overall, the faculty facilitators felt the TBL stimulated high-quality discussion, during which students remained engaged and asked thought-provoking questions. This was very significant as the students were clearly stressed about the impending course final exam and USMLE Step 1. This meaningful dialogue was observed each year the TBL was delivered. Faculty members also performed a qualitative analysis of course evaluations in an effort to gain a better understanding of student perceptions and opinions of the TBL. Thematic analysis was completed by independent reviewers. In general, students provided many positive comments about the TBL. For example, students commented that the TBL was interesting, relevant, and engaging and helped integrate and solidify information to prepare them for the psychiatry clerkship. They felt the content pushed them to think critically and the discussions covered material considered very applicable for their future careers, such as how to assess risk factors for suicide.

## Discussion

According to the World Health Organization, suicide is considered a global phenomenon, with one person dying from suicide every 40 seconds, totaling close to 800,000 deaths every year.^32^ In addition, many more people attempt suicide on a daily basis. Although suicide is considered a worldwide social and medical problem, studies have shown that health care professionals and students are not given adequate training for SAM.^33,34^ For this reason, the availability of educational materials, especially those utilizing more active learning instructional strategies, is of utmost importance.

This TBL module, which covers SAM, is a valuable resource that can be used to enhance education on this important topic through student interaction and discussion. It not only addresses the importance of suicide risk assessment but also covers acute and long-term treatment strategies required in the clinical management of at-risk patients. Throughout the TBL, students are required to critically evaluate and discuss real-life scenarios, helping prepare them for difficult situations they may encounter in a clinical setting. Although developed for second-year medical students, the module can be used or modified for the education of other health professionals as well. Overall, this interactive resource can provide health profession educators with an opportunity to fill in the gaps in their students' training about suicide prevention and management.

The faculty involved in the development and improvement of the TBL utilized guidelines and tips from literature-based resources to create a valuable module. The TBL was designed over a period of several months. One important recommendation was the use of backward design, which focused on what students should be able to accomplish at the end of the TBL, rather than on what students should know.^16^ By using backward design, the TBL could offer an opportunity for students to not only master content but also learn to apply it.

In keeping with the recommendations of backward design, the first step involved developing specific aims and learning outcomes, during which we considered the intentions for the module and determined what students should be able to do at the end of the activity. Once the aims and outcomes were developed, the application exercise questions were created to promote higher-level critical thinking and engagement. They adhered to the 4 S's of TBL design (i.e., significant problem, same problem, specific choice, simultaneous report). Lastly, the advance reading materials were chosen and RAT questions developed to make sure students read the required preparatory assignment. Throughout its development, faculty members met several times and communicated through email. Overall, several renditions of application questions and RAT questions were completed prior to finalizing the module. In addition, some questions were modified after implementation in an effort to improve the TBL for the following year.

One of the strengths utilized in developing this TBL was the diverse skill sets of the contributing faculty. For example, faculty with expertise in psychiatry, neurology, and pharmacology assisted with the development of the module, allowing for diverse perspectives and integration of material. Additionally, several of the faculty members were certified TBLC trainer-consultants, considered expert educators and consultants for those interested in implementing TBL. For this reason, these faculty members were well equipped to assist other faculty with the creation, improvement, and facilitation of the TBL activity.

Major strengths of the TBL were the valuable debates and discussions that were generated throughout the entire session. Students asked and answered challenging questions, clearly displaying their use of critical thinking and clinical reasoning. The facilitation of the TBL was also a strength: Limited time was spent on lecturing and sharing faculty opinions, allowing students to lead the discussion and answer questions. Open-ended questions were also consistently asked in follow-up to the application exercise questions. In addition, all teams were encouraged to participate in a respectful manner, and the answers were never revealed until a thorough discussion had taken place.

Although the TBL was considered a successful addition to the psychopathology course, there were a few important considerations for improvement to create a more effective learning environment. At OUWB, psychopathology was a 2-week course occurring in the second year of the curriculum. It was also the last organ-system course prior to the extremely important Step 1 licensure exam. For these reasons, it was challenging to schedule the TBL in a way that allowed students enough time to prepare while not adding to the stress they were already experiencing. We wanted to make sure students were given sufficient time to learn the basic science concepts in order to apply the material during the application portion of the TBL module.

The first year we offered this TBL, it was scheduled at the end of the course, just prior to the final exam. Some students felt this was too close to the exam. For this reason, it was moved to the beginning of the course, when over half of the course material had not yet been delivered. According to course evaluations, students felt unprepared to answer the application questions to the best of their ability due to the timing. After further evaluation, both students and faculty felt strongly that the TBL would fit more appropriately at the end of the course, after students had the opportunity to study all materials. Placing the TBL module at the end of the course gave students more time to absorb and digest all of the different concepts presented and allowed them to better synthesize and apply information during the TBL itself.

Another lesson learned was the importance of communication with the students. Based on our experiences as medical educators, medical students tend to want answers to be black and white and are much more uncomfortable when questions and answers contain shades of grey. TBL application activities are often meant to be complicated clinical scenarios, but compared to other TBL activities throughout the curriculum, this TBL module's application exercises are particularly complex in nature. Another lesson learned was the need to remind students that the application questions would be difficult, that there might be more than one correct answer, and that the answers could be somewhat subjective. Unfortunately, in health care, patient problems are never black and white. For this reason, it was important to stress to students that often, when taking care of patients, the answers may not be clear and there may be more than one appropriate way to address a difficult situation. Even the most knowledgeable and skilled clinicians can reach different conclusions when evaluating the same patient. Overall, while this TBL was meant to educate students about SAM, it was also important that students realized the TBL was designed to help them become more comfortable with the ambiguity they will often experience during patient assessment and clinical decision-making. It may be best to communicate this to students prior to the TBL, to help them feel more comfortable when they arrive at a different answer than their peers or the TBL facilitators.

In addition to the lessons learned, there are a few additional opportunities to consider for revision in order to make the TBL more beneficial. One potential modification could be changing the preparatory assignment. Simon and Hales' *The American Psychiatric Publishing Textbook of Suicide Assessment and Management* is an excellent resource that covers the challenges clinicians face in assessing and managing suicide risk and preventing tragedy.^25^ That said, some students felt the assigned reading was choppy and difficult to understand. Others who implement this TBL may consider using a different preparatory assignment, which could include another journal article or even material from a mini-lecture.

Another possible area for improvement could be to update some of the RAT and application questions. The RAT questions could be modified to short patient cases that help ensure students have a comprehensive understanding of the foundational concepts. The application exercises could be modified to make them less complex and vague. While it is important that students learn to be comfortable with ambiguity, in some cases no team chose the best answer (e.g., Appendix D, case questions 1 and 3). Although students felt the TBL was valuable, this could induce frustration among learners. It may be helpful to make some of the questions less challenging, especially depending on the level of the learners. In addition, it may be more effective to leave some of the questions open-ended, which could give the TBL facilitator a better idea of the students' thought process, as well as their ideas and feelings. Lastly, it may be helpful to develop a new case addressing suicide risk assessment with an elderly individual as this could help learners develop a better sense of the developmental course by contrasting an adolescent with an older adult.

### Conclusion

Suicide is a global health problem that health care providers must feel comfortable addressing. Unfortunately, many health care providers do not feel equipped to assess and treat patients at risk for suicide due to lack of training and education. Overall, we believe this SAM TBL offers health professions educators the opportunity to utilize an interactive resource that addresses a gap in suicide education. This TBL not only teaches students the fundamentals of suicide assessment and prevention; it also provides them with important skills that are necessary for more effective interactions with patients, thereby promoting successful outcomes.

## Appendices

Student Handout.docxReadiness Assurance Test Template.docxAppeal Form.docxPowerPoint Presentation Template.pptxReadiness Assurance Test Response Rates.docxApplication Exercise Response Rates.docxApplication Exercise Explanations.docx
All appendices are peer reviewed as integral parts of the Original Publication.
