# Supplementary material for: Suicide Assessment and Management Team-Based Learning Module
Source: MedEdPORTAL. 2020 Aug 20;16:10952. doi: 10.15766/mep_2374-8265.10952 (PMC7449577; doi:10.15766/mep_2374-8265.10952)
Supplement: Supplementary file 1 — Student Handout.docxReadiness Assurance Test Template.docxAppeal Form.docxPowerPoint Presentation Template.pptxReadiness Assurance Test Response Rates.docxApplication Exercise Response Rates.docxApplication Exercise Explanations.docx [file mep_2374-8265.10952-s001.zip › A. Student Handout.docx]

**Suicide Assessment and Management TBL**

***Student Handout***

**Educational Objectives:**

By the end of this activity, learners will be able to:

1. Identify risk factors associated with suicide.
2. Compare and contrast subgroups of the population that are at increased risk for suicide, including children, adolescents, and elderly individuals and discuss how to eliminate disparities among these vulnerable groups.
3. Indicate which medications are used to reduce the risk of suicide and which are used in the clinical management of a suicidal individual.
4. Discuss the key elements that must be evaluated in order to conduct a comprehensive assessment of a patient’s risk for suicide.
5. Compare the major treatment strategies (acute and long term) utilized to optimally treat patients who are assessed and believed to be at significant risk for suicide.
6. Create a clinical management plan for a suicidal patient using evidence-based medicine.
7. Discuss differences in state policies for efficient and effective continuity of care.
8. Participate in the TBL activity in a professional and respectful manner.
9. Engage the material by critically evaluating its content and employing peer teaching throughout the session.

**Preparatory Reading Assignment**:

Simon R, Hales R. *The American Psychiatric Publishing Textbook of Suicide Assessment and Management*. 2^nd^ ed. Arlington, VA: American Psychiatric Publishing; 2012.

1. Chapter 1: Suicide Risk Assessment: Gateway to Treatment and Management.  Pages 12-21 (from Systematic Suicide Risk Assessment through Populations at Risk for Suicide)
2. Chapter 12: Psychopharmacotherapy and Electroconvulsive Therapy. Pages 216-218 and 222-226 (Pharmacological Treatment through Electroconvulsive Therapy)
3. Chapter 20: Children, Adolescents, and College Students.  Page 350 - Table 20.1.
4. Chapter 21: The Elderly.  Pages 378-379 (Management of Suicide Risk in Late Life - Assessment and Intervention through Prevention of Suicide in Late Life).

**Session Activity will include:**

1. iRAT/tRAT
2. Intersession Discussion
3. Application Exercise
