# Supplementary material for: Suicide Assessment and Management Team-Based Learning Module
Source: MedEdPORTAL. 2020 Aug 20;16:10952. doi: 10.15766/mep_2374-8265.10952 (PMC7449577; doi:10.15766/mep_2374-8265.10952)
Supplement: Supplementary file 1 — Student Handout.docxReadiness Assurance Test Template.docxAppeal Form.docxPowerPoint Presentation Template.pptxReadiness Assurance Test Response Rates.docxApplication Exercise Response Rates.docxApplication Exercise Explanations.docx [file mep_2374-8265.10952-s001.zip › B. Readiness Assurance Test Template.docx]

ATTENTION, STUDENTS: If you are accessing this material BEFORE it is used in your course, please do NOT read this document prior to the class session. An answer key is included in this module, which is designed to lead you through a learning experience that reinforces your knowledge of the content. Early review or dissemination of this material to others will diminish the learning opportunity and be considered academic misconduct.

**Course:**

**Suicide Assessment and Management TBL**

**Date:**

**Readiness Assurance Test (iRAT/tRAT)**

1. Suicide risk is reliably predicted by which *single* factor?
   1. Family history of mood disorders
   2. Past suicide attempt
   3. Persistent feelings of hopelessness
   4. None of the above
2. What demographic has the *highest* suicide rate?
   1. African American males between 12-15 years of age
   2. Hispanic females between 15-18 years of age
   3. White females between 65-70 years of age
   4. White males between 85-90 years of age
3. What is a *key difference* between suicidal adolescents and suicidal adults?
   1. Contagion effects are more powerful in suicidal adults.
   2. Serotonin reuptake inhibitors require more monitoring in suicidal adolescents.
   3. Suicide attempts are more common in suicidal adults.
   4. Suicidal ideation is less likely to be denied when asked about in suicidal adolescents.
4. Which antipsychotic medication is indicated for suicide risk reduction in patients with schizophrenia?
   1. Clozapine
   2. Olanzapine
   3. Risperidone
   4. Thioridazine
5. Suicide is the leading cause of death among persons younger than 35 years of age with which psychiatric diagnosis?
6. Attention deficit hyperactivity disorder
7. Bipolar disorder
8. Depression
9. Schizophrenia

**Answers**

1. d (None of the above)
2. d (White males between 85-90 years of age)
3. b (Serotonin reuptake inhibitors require *more* monitoring in suicidal *adolescents*)
4. a (Clozapine)
5. d (Schizophrenia)
