# Supplementary material for: Suicide Assessment and Management Team-Based Learning Module
Source: MedEdPORTAL. 2020 Aug 20;16:10952. doi: 10.15766/mep_2374-8265.10952 (PMC7449577; doi:10.15766/mep_2374-8265.10952)
Supplement: Supplementary file 1 — Student Handout.docxReadiness Assurance Test Template.docxAppeal Form.docxPowerPoint Presentation Template.pptxReadiness Assurance Test Response Rates.docxApplication Exercise Response Rates.docxApplication Exercise Explanations.docx [file mep_2374-8265.10952-s001.zip › C. Appeal Form.docx]

**TBL APPEAL FORM*:*** *The appeal must be submitted to [INSERT EMAIL] within 24 hours after the TBL session. Appeals must be submitted AFTER the TBL activity. Forms submitted during the session will not be reviewed.*

Team Number:

Team Member Names:

1.

2.

3.

4.

5.

6.

7.

TBL Session:

tRAT question #:

Describe the issue you found with the question (e.g. wording or content). Please make sure to reference the material upon which your argument is based:

Faculty Response to Appeal:
