# Supplementary material for: Suicide Assessment and Management Team-Based Learning Module
Source: MedEdPORTAL. 2020 Aug 20;16:10952. doi: 10.15766/mep_2374-8265.10952 (PMC7449577; doi:10.15766/mep_2374-8265.10952)
Supplement: Supplementary file 1 — Student Handout.docxReadiness Assurance Test Template.docxAppeal Form.docxPowerPoint Presentation Template.pptxReadiness Assurance Test Response Rates.docxApplication Exercise Response Rates.docxApplication Exercise Explanations.docx [file mep_2374-8265.10952-s001.zip › D. PowerPoint Presentation Template.pptx]

## Slide 1
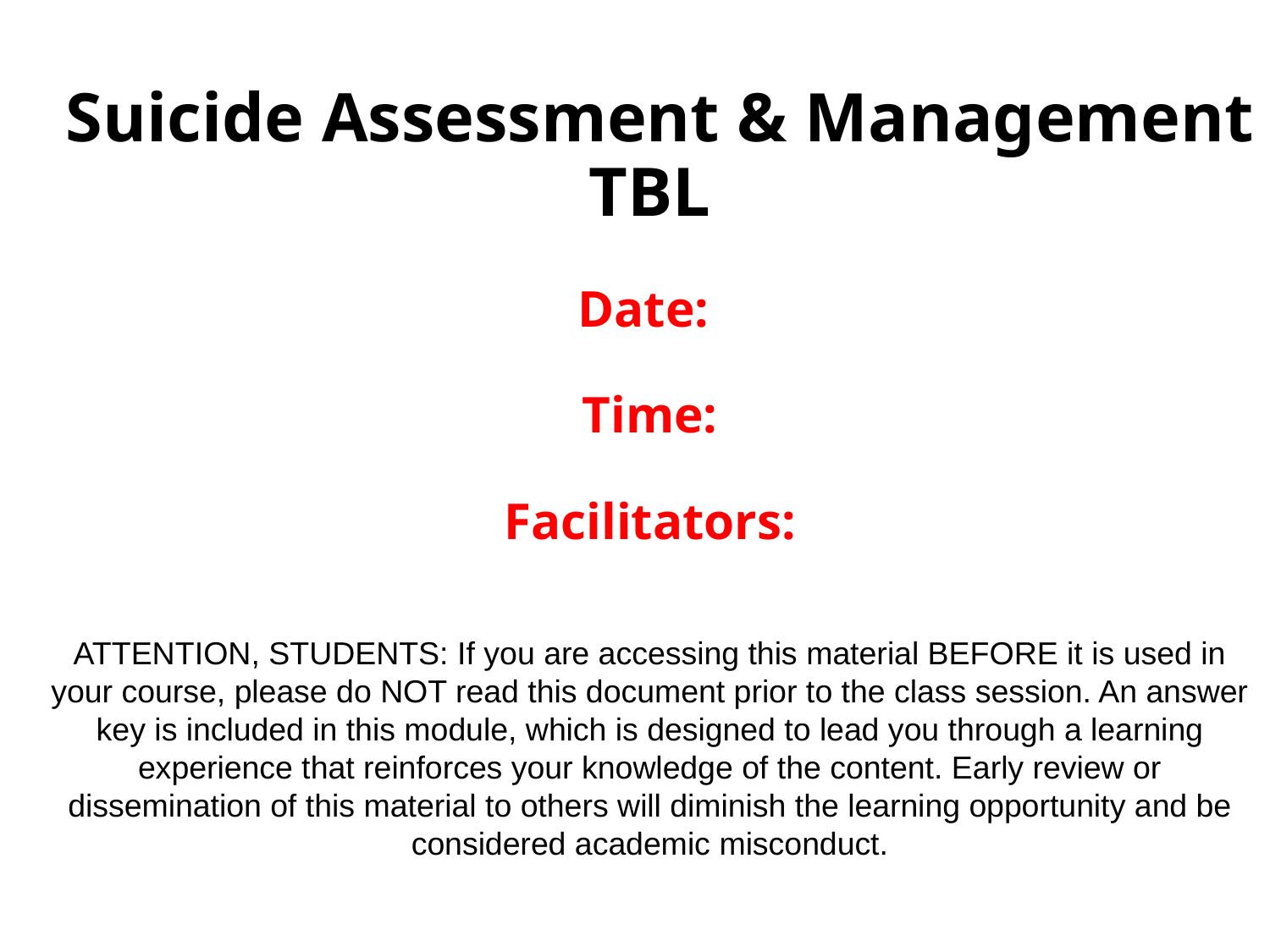

# Suicide Assessment & Management TBL Date: Time: Facilitators:
ATTENTION, STUDENTS: If you are accessing this material BEFORE it is used in your course, please do NOT read this document prior to the class session. An answer key is included in this module, which is designed to lead you through a learning experience that reinforces your knowledge of the content. Early review or dissemination of this material to others will diminish the learning opportunity and be considered academic misconduct.

## Slide 2
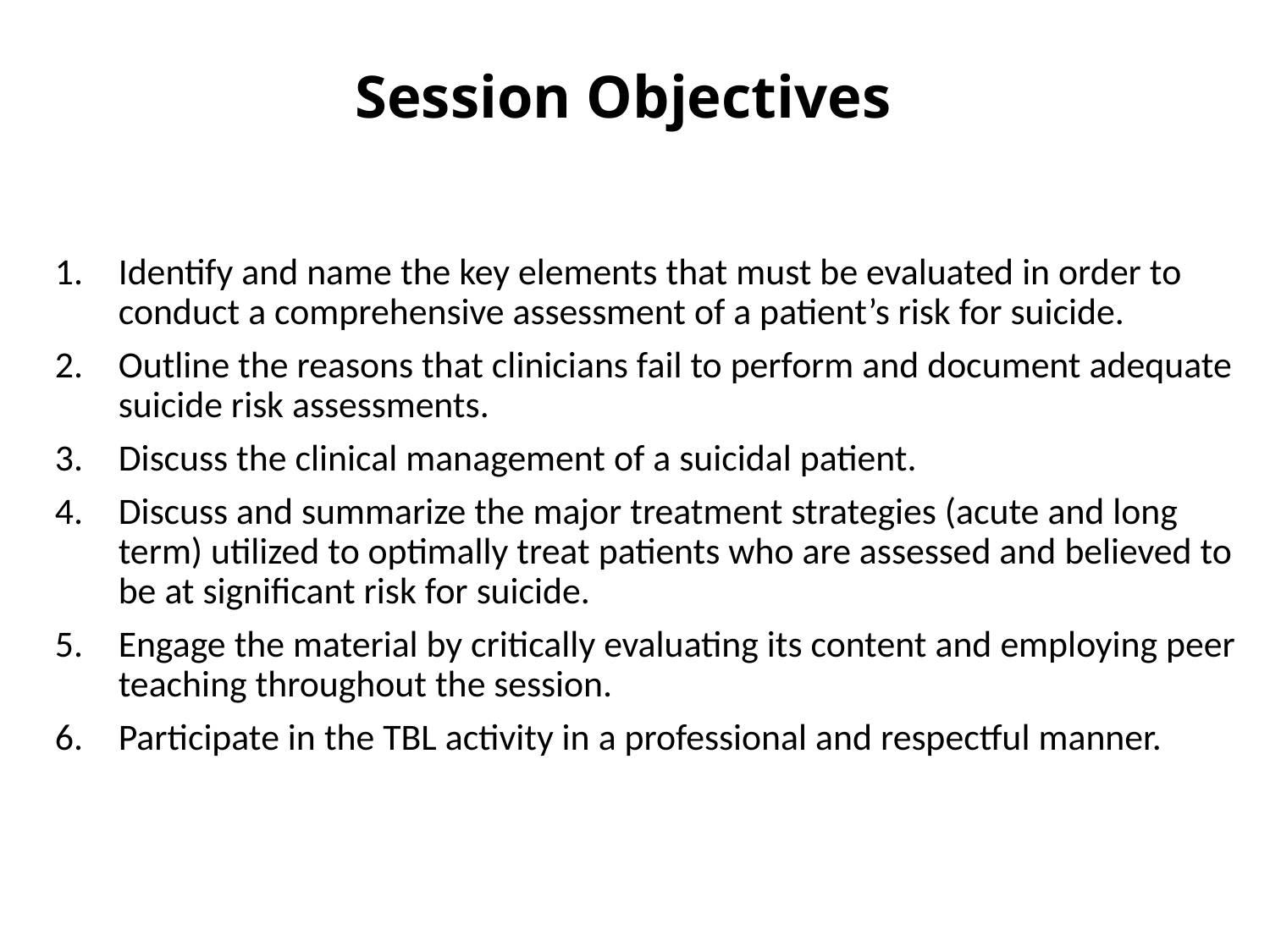

# Session Objectives
Identify and name the key elements that must be evaluated in order to conduct a comprehensive assessment of a patient’s risk for suicide.
Outline the reasons that clinicians fail to perform and document adequate suicide risk assessments.
Discuss the clinical management of a suicidal patient.
Discuss and summarize the major treatment strategies (acute and long term) utilized to optimally treat patients who are assessed and believed to be at significant risk for suicide.
Engage the material by critically evaluating its content and employing peer teaching throughout the session.
Participate in the TBL activity in a professional and respectful manner.

## Slide 3
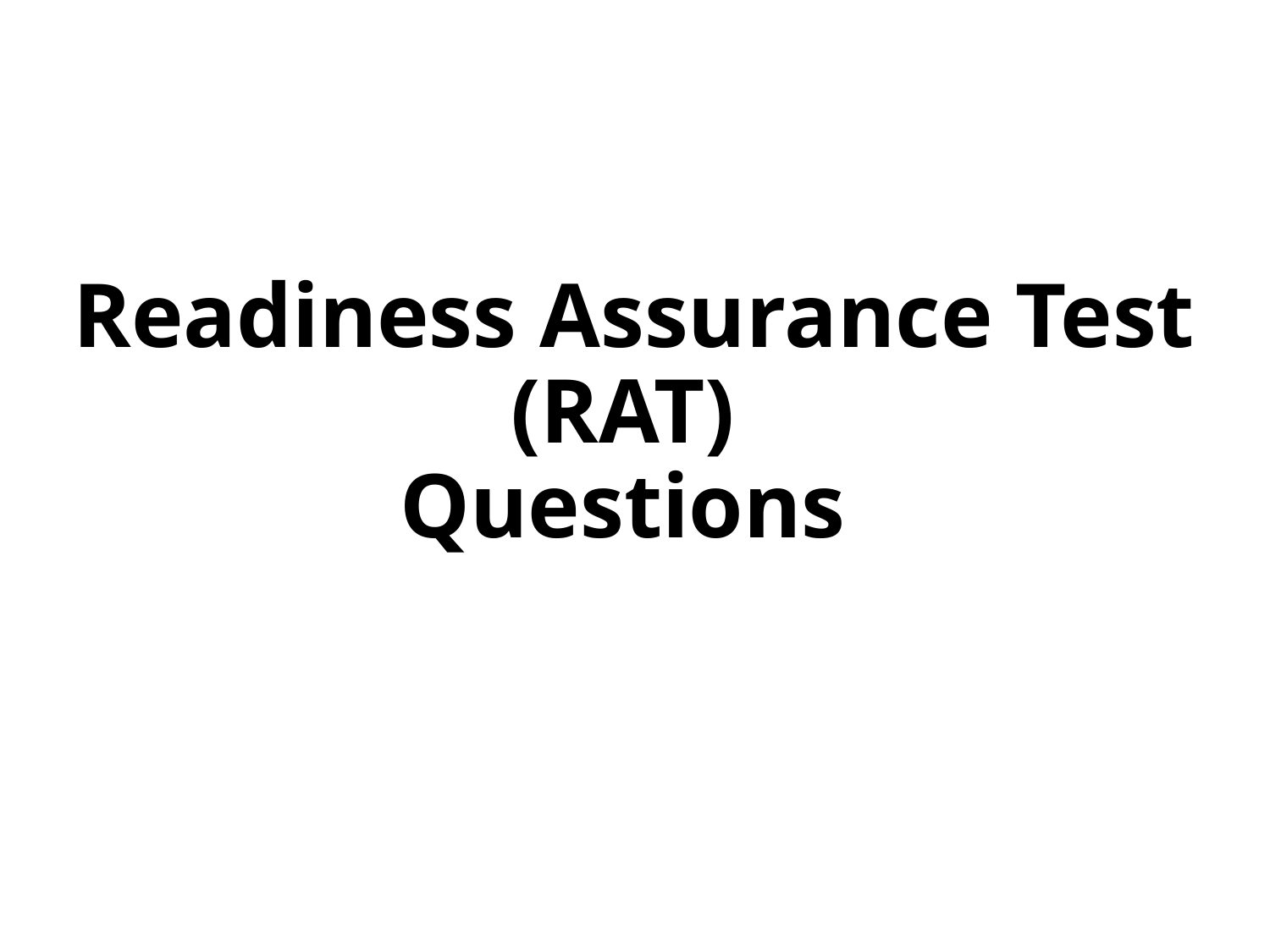

# Readiness Assurance Test (RAT) Questions

## Slide 4
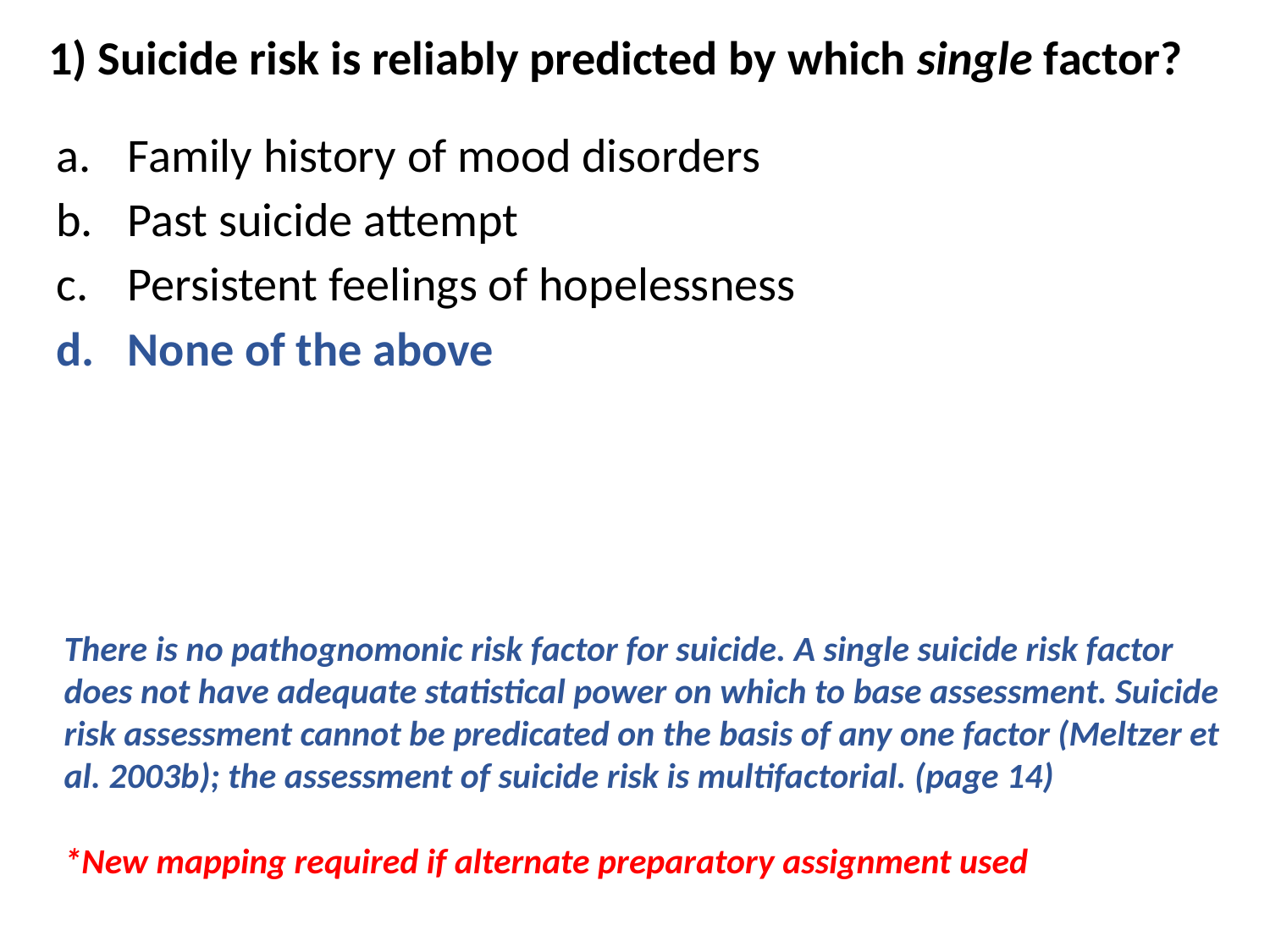

1) Suicide risk is reliably predicted by which single factor?
Family history of mood disorders
Past suicide attempt
Persistent feelings of hopelessness
None of the above
There is no pathognomonic risk factor for suicide. A single suicide risk factor does not have adequate statistical power on which to base assessment. Suicide risk assessment cannot be predicated on the basis of any one factor (Meltzer et al. 2003b); the assessment of suicide risk is multifactorial. (page 14)
*New mapping required if alternate preparatory assignment used

## Slide 5
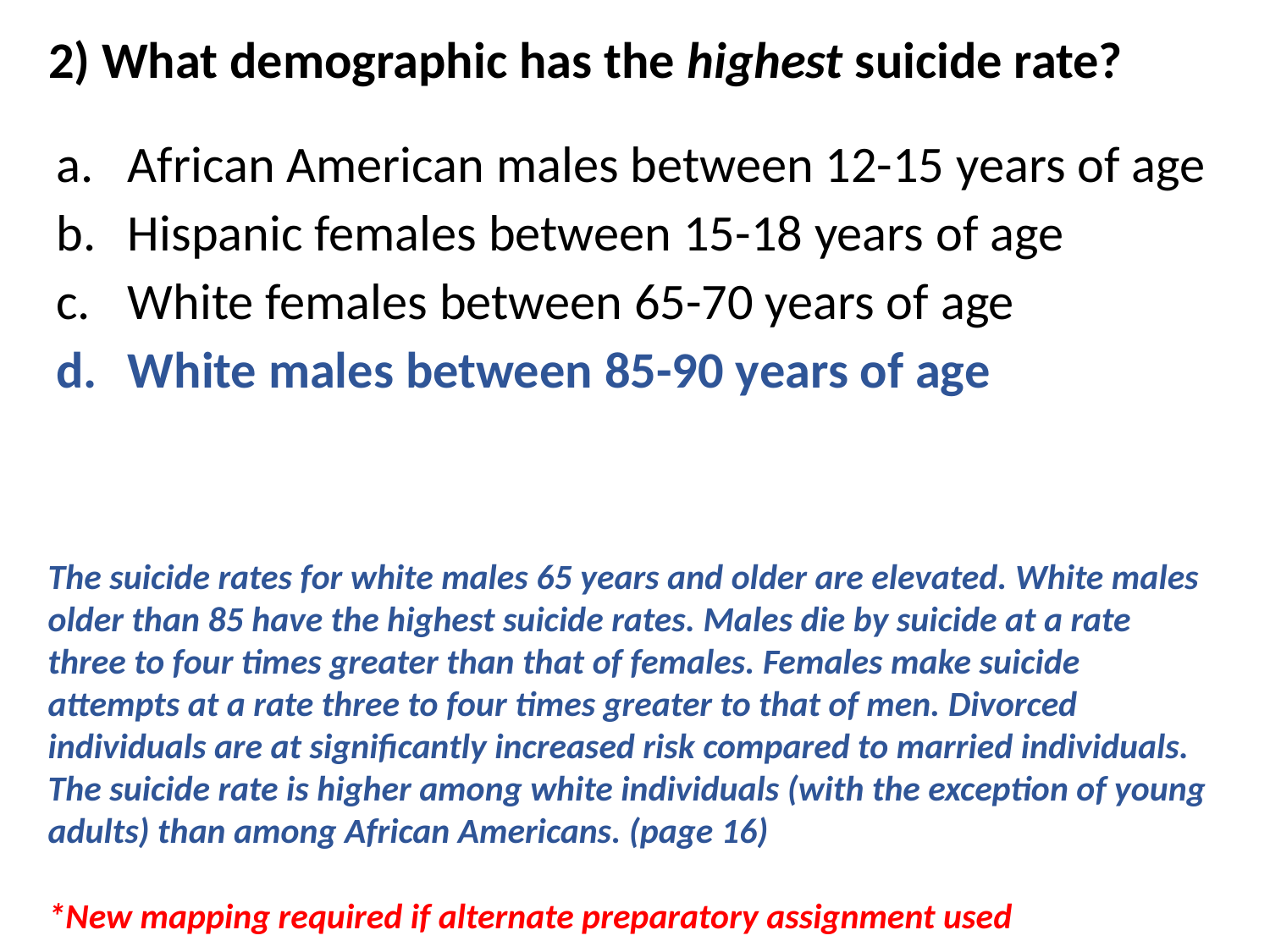

2) What demographic has the highest suicide rate?
African American males between 12-15 years of age
Hispanic females between 15-18 years of age
White females between 65-70 years of age
White males between 85-90 years of age
The suicide rates for white males 65 years and older are elevated. White males older than 85 have the highest suicide rates. Males die by suicide at a rate three to four times greater than that of females. Females make suicide attempts at a rate three to four times greater to that of men. Divorced individuals are at significantly increased risk compared to married individuals. The suicide rate is higher among white individuals (with the exception of young adults) than among African Americans. (page 16)
*New mapping required if alternate preparatory assignment used

## Slide 6
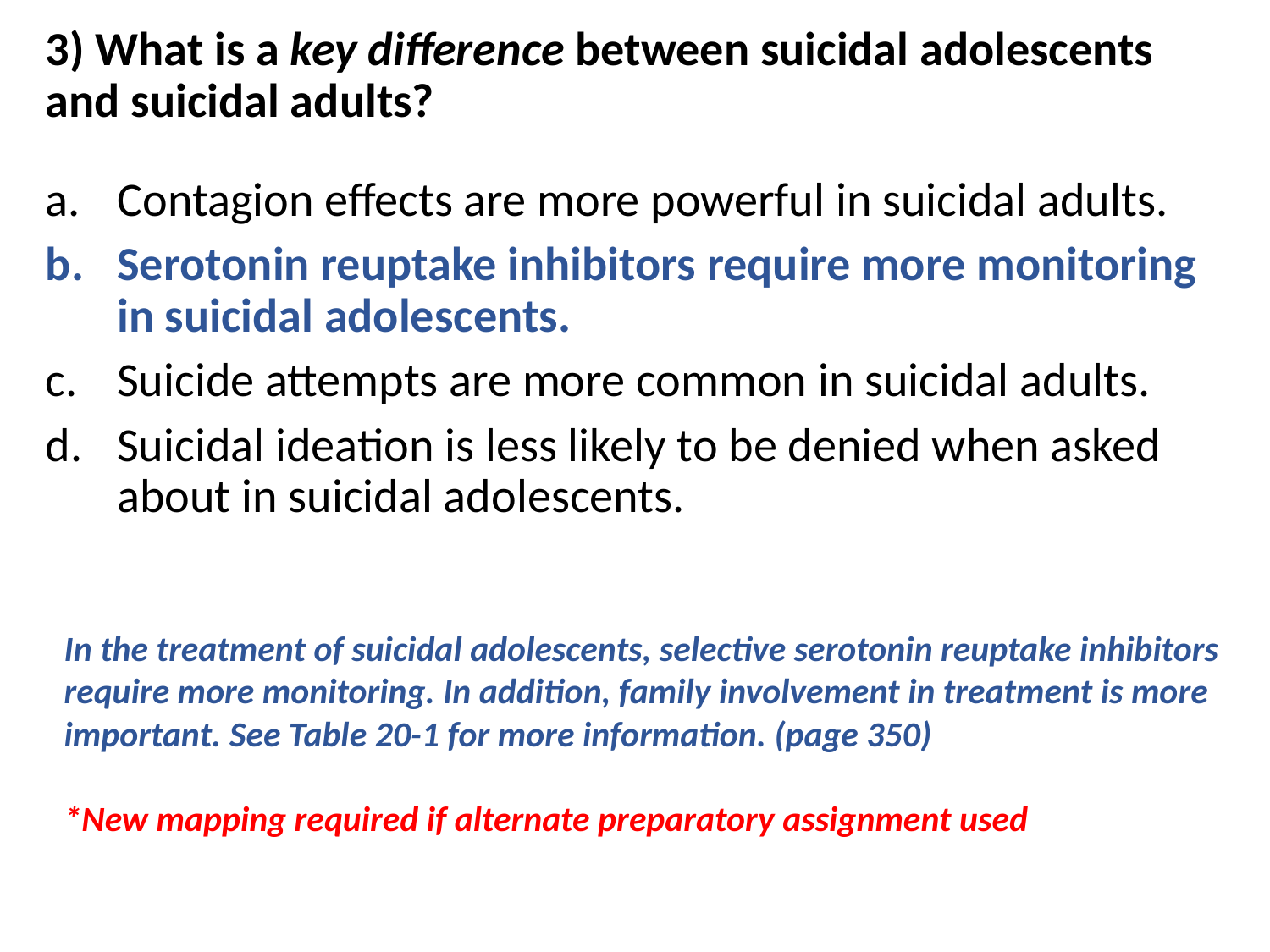

3) What is a key difference between suicidal adolescents and suicidal adults?
Contagion effects are more powerful in suicidal adults.
Serotonin reuptake inhibitors require more monitoring in suicidal adolescents.
Suicide attempts are more common in suicidal adults.
Suicidal ideation is less likely to be denied when asked about in suicidal adolescents.
In the treatment of suicidal adolescents, selective serotonin reuptake inhibitors require more monitoring. In addition, family involvement in treatment is more important. See Table 20-1 for more information. (page 350)
*New mapping required if alternate preparatory assignment used

## Slide 7
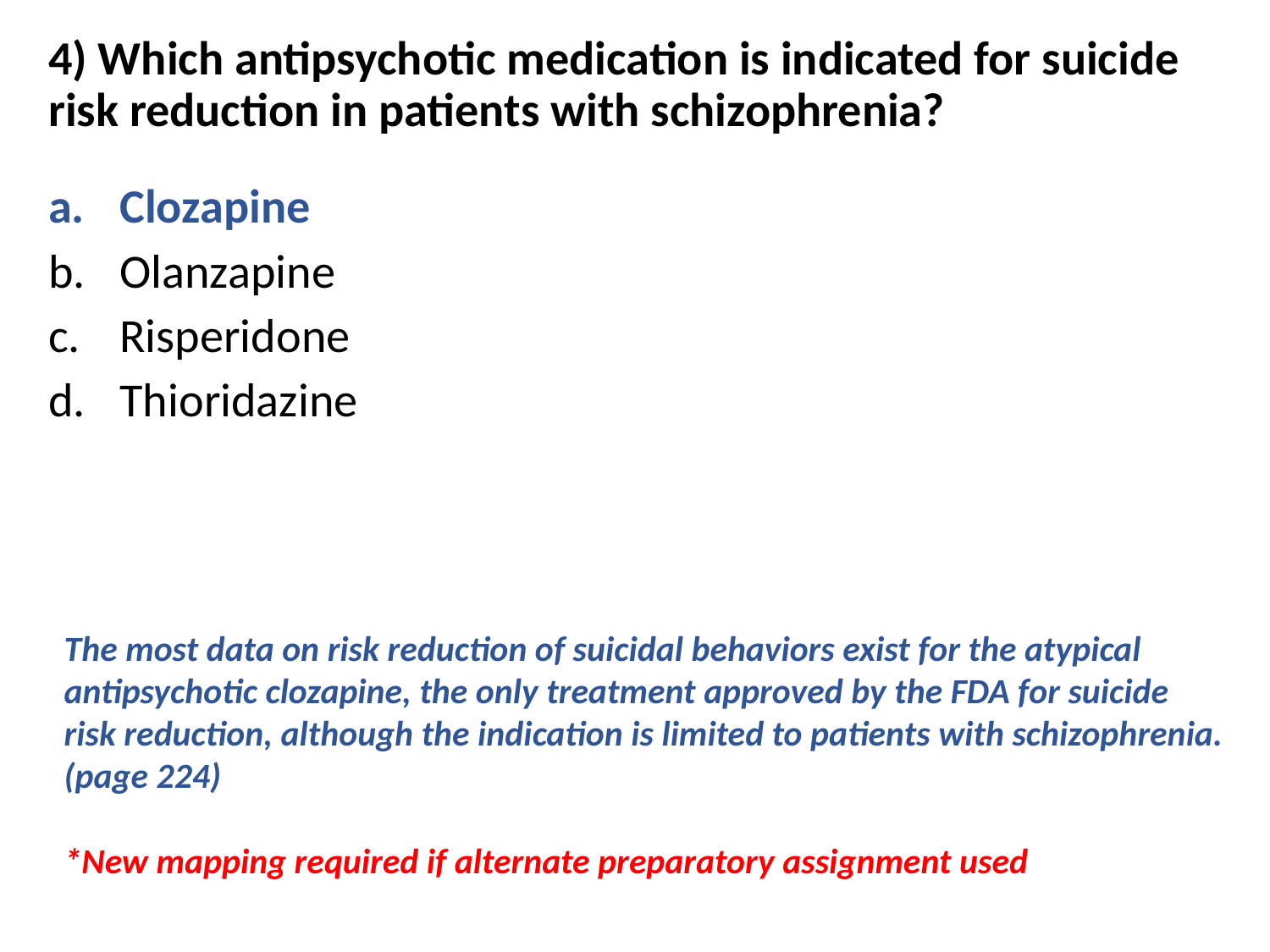

4) Which antipsychotic medication is indicated for suicide risk reduction in patients with schizophrenia?
Clozapine
Olanzapine
Risperidone
Thioridazine
The most data on risk reduction of suicidal behaviors exist for the atypical antipsychotic clozapine, the only treatment approved by the FDA for suicide risk reduction, although the indication is limited to patients with schizophrenia. (page 224)
*New mapping required if alternate preparatory assignment used

## Slide 8
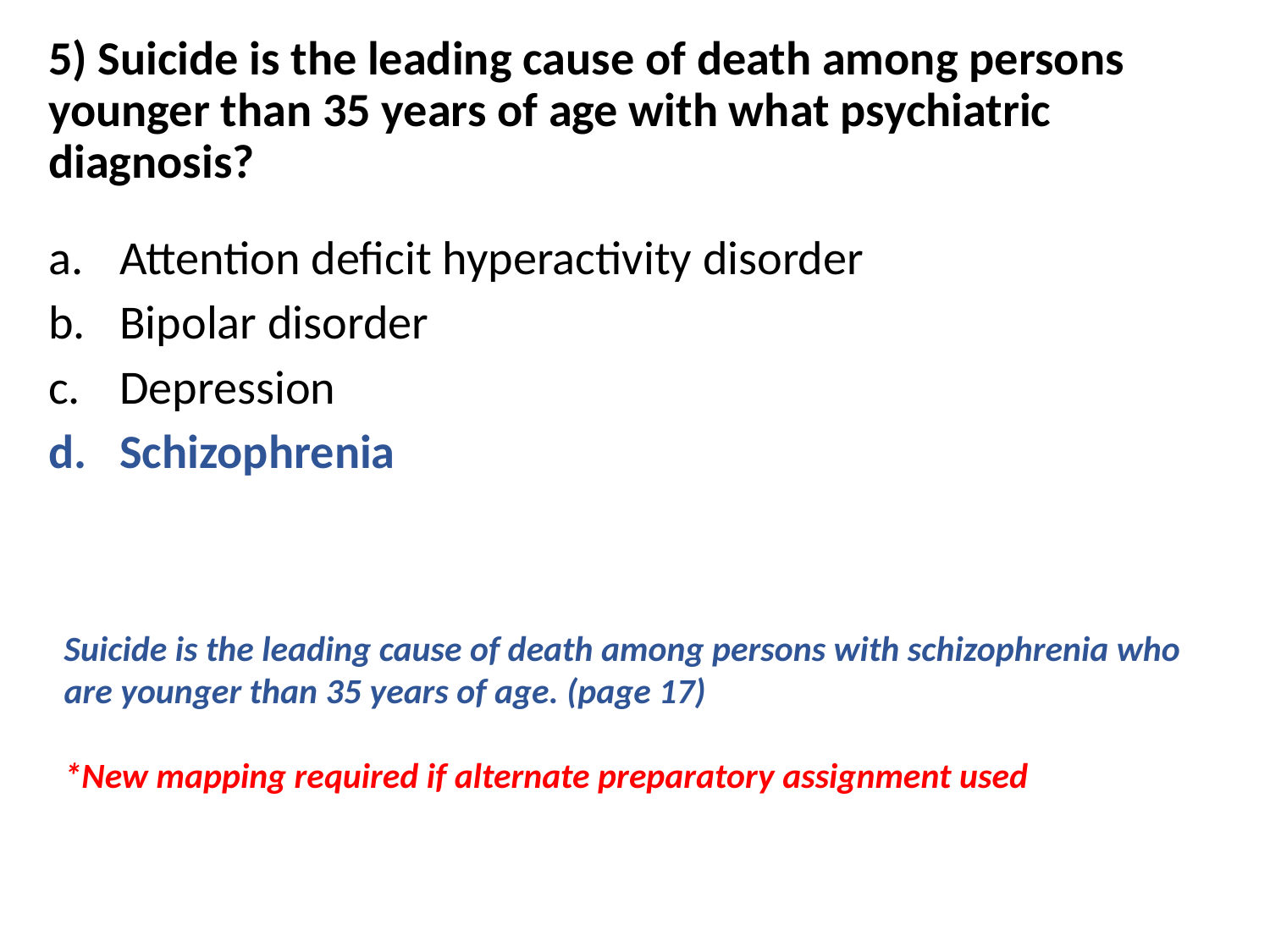

5) Suicide is the leading cause of death among persons younger than 35 years of age with what psychiatric diagnosis?
Attention deficit hyperactivity disorder
Bipolar disorder
Depression
Schizophrenia
Suicide is the leading cause of death among persons with schizophrenia who are younger than 35 years of age. (page 17)
*New mapping required if alternate preparatory assignment used

## Slide 9
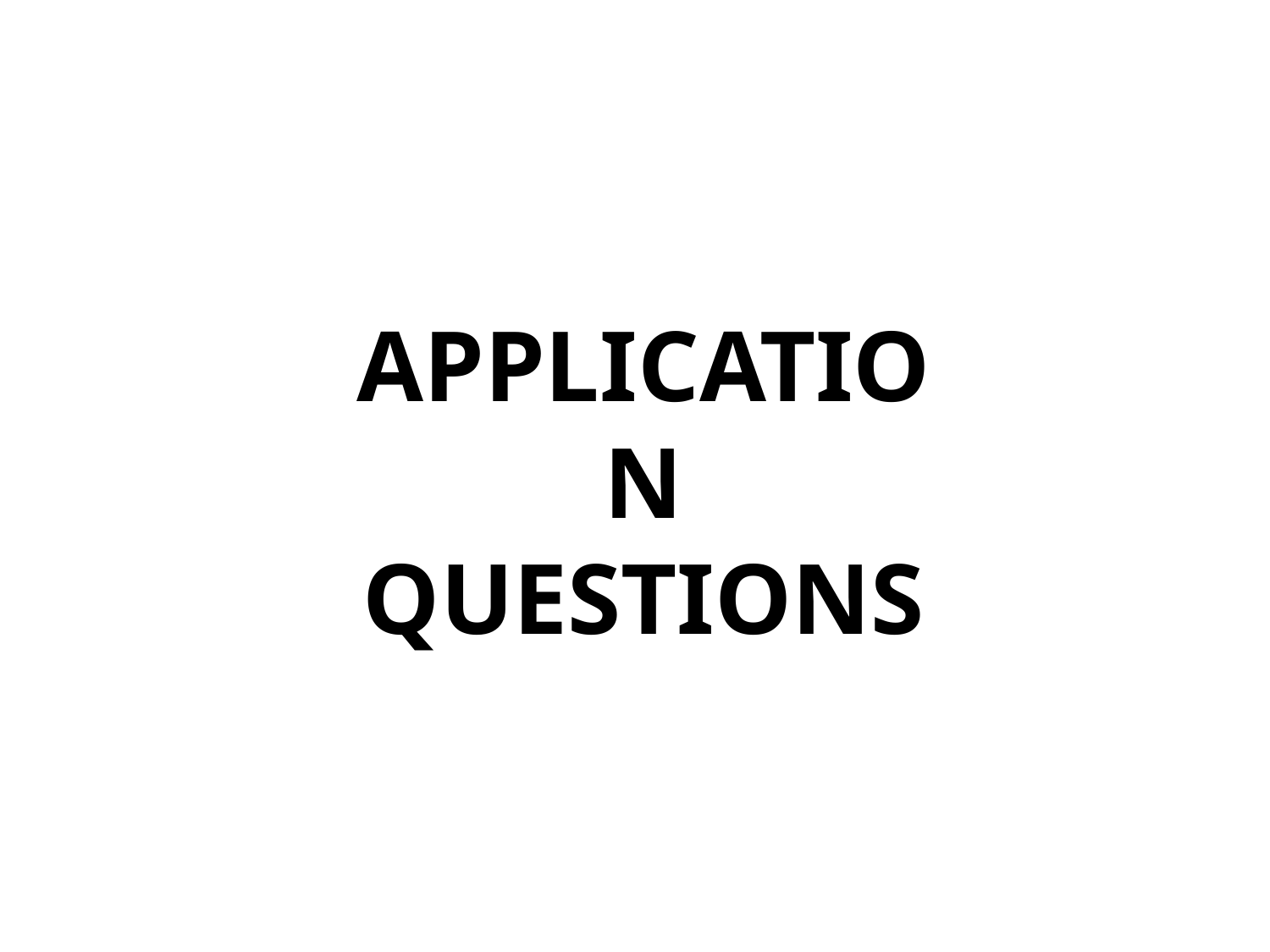

APPLICATION QUESTIONS

## Slide 10
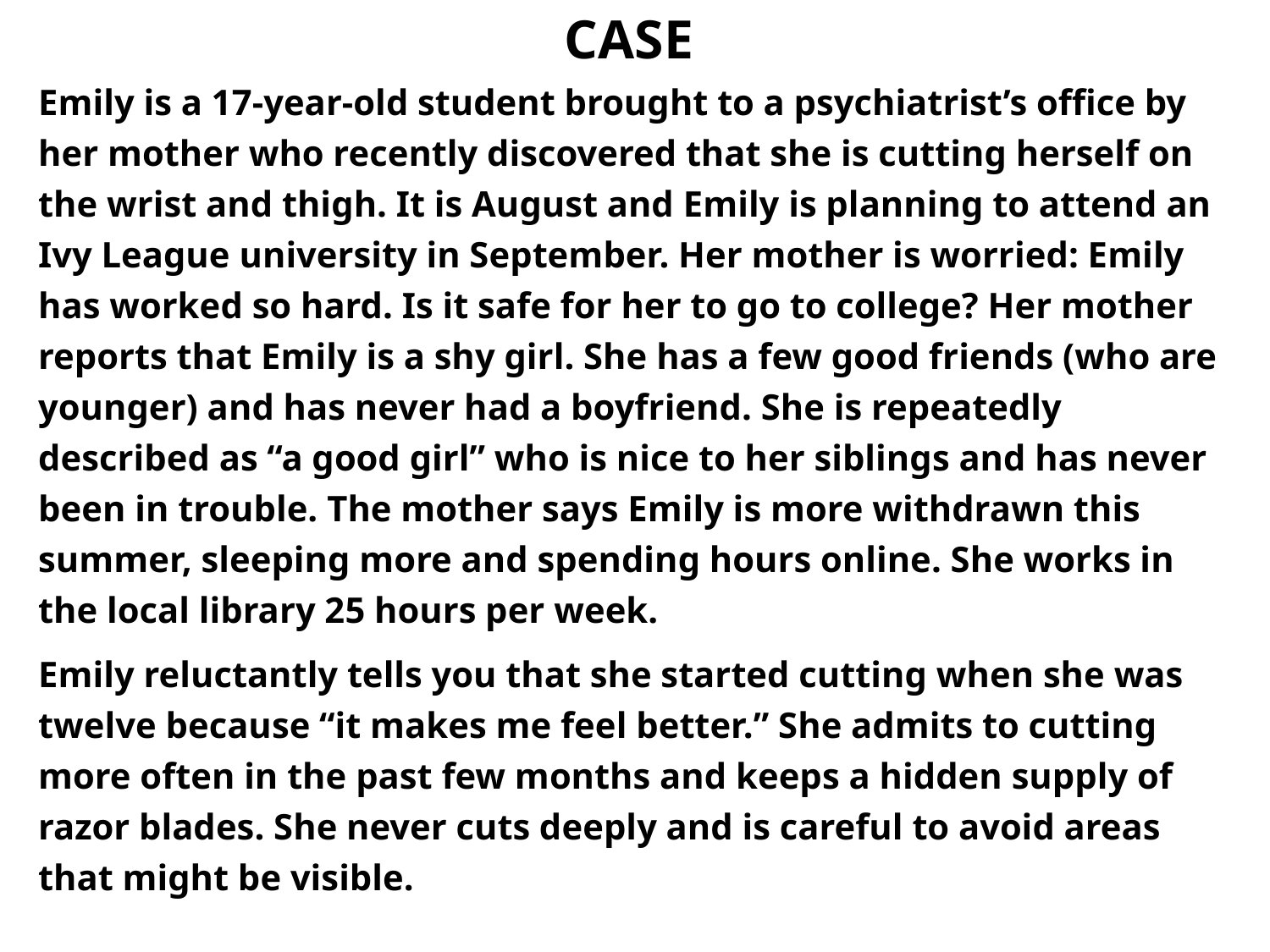

CASE
Emily is a 17-year-old student brought to a psychiatrist’s office by her mother who recently discovered that she is cutting herself on the wrist and thigh. It is August and Emily is planning to attend an Ivy League university in September. Her mother is worried: Emily has worked so hard. Is it safe for her to go to college? Her mother reports that Emily is a shy girl. She has a few good friends (who are younger) and has never had a boyfriend. She is repeatedly described as “a good girl” who is nice to her siblings and has never been in trouble. The mother says Emily is more withdrawn this summer, sleeping more and spending hours online. She works in the local library 25 hours per week.
Emily reluctantly tells you that she started cutting when she was twelve because “it makes me feel better.” She admits to cutting more often in the past few months and keeps a hidden supply of razor blades. She never cuts deeply and is careful to avoid areas that might be visible.

## Slide 11
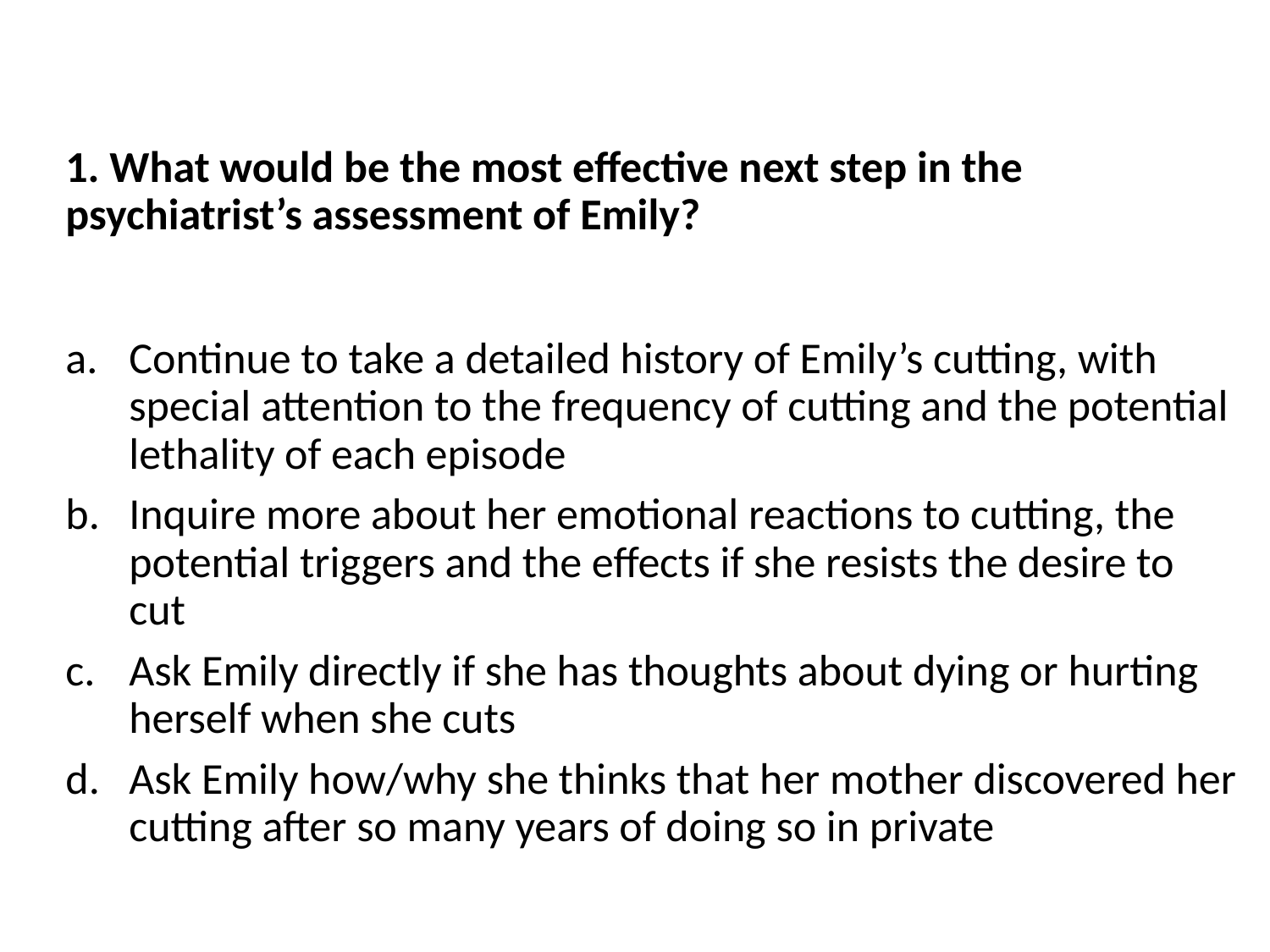

1. What would be the most effective next step in the psychiatrist’s assessment of Emily?
Continue to take a detailed history of Emily’s cutting, with special attention to the frequency of cutting and the potential lethality of each episode
Inquire more about her emotional reactions to cutting, the potential triggers and the effects if she resists the desire to cut
Ask Emily directly if she has thoughts about dying or hurting herself when she cuts
Ask Emily how/why she thinks that her mother discovered her cutting after so many years of doing so in private

## Slide 12
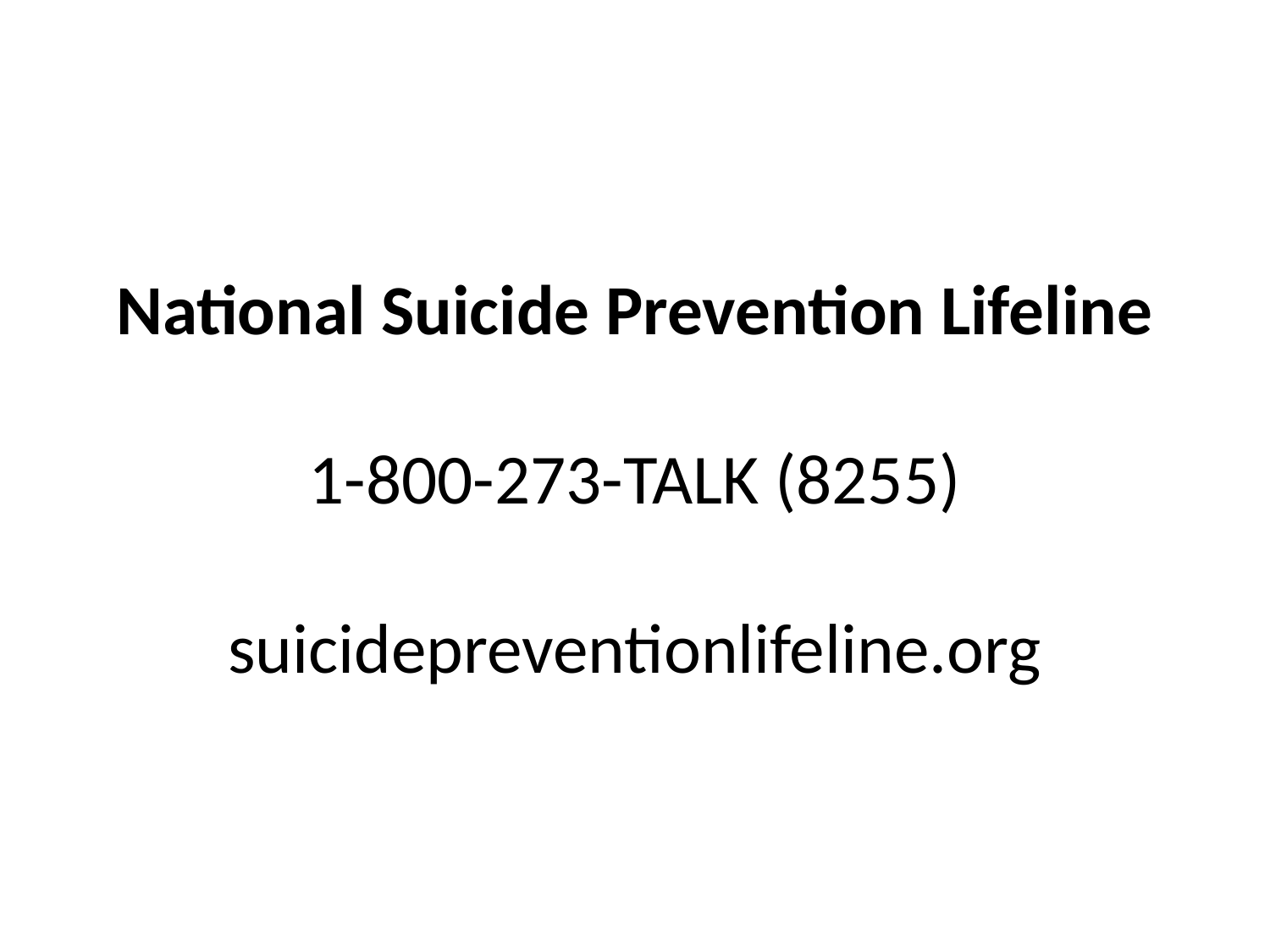

National Suicide Prevention Lifeline
1-800-273-TALK (8255)
suicidepreventionlifeline.org

## Slide 13
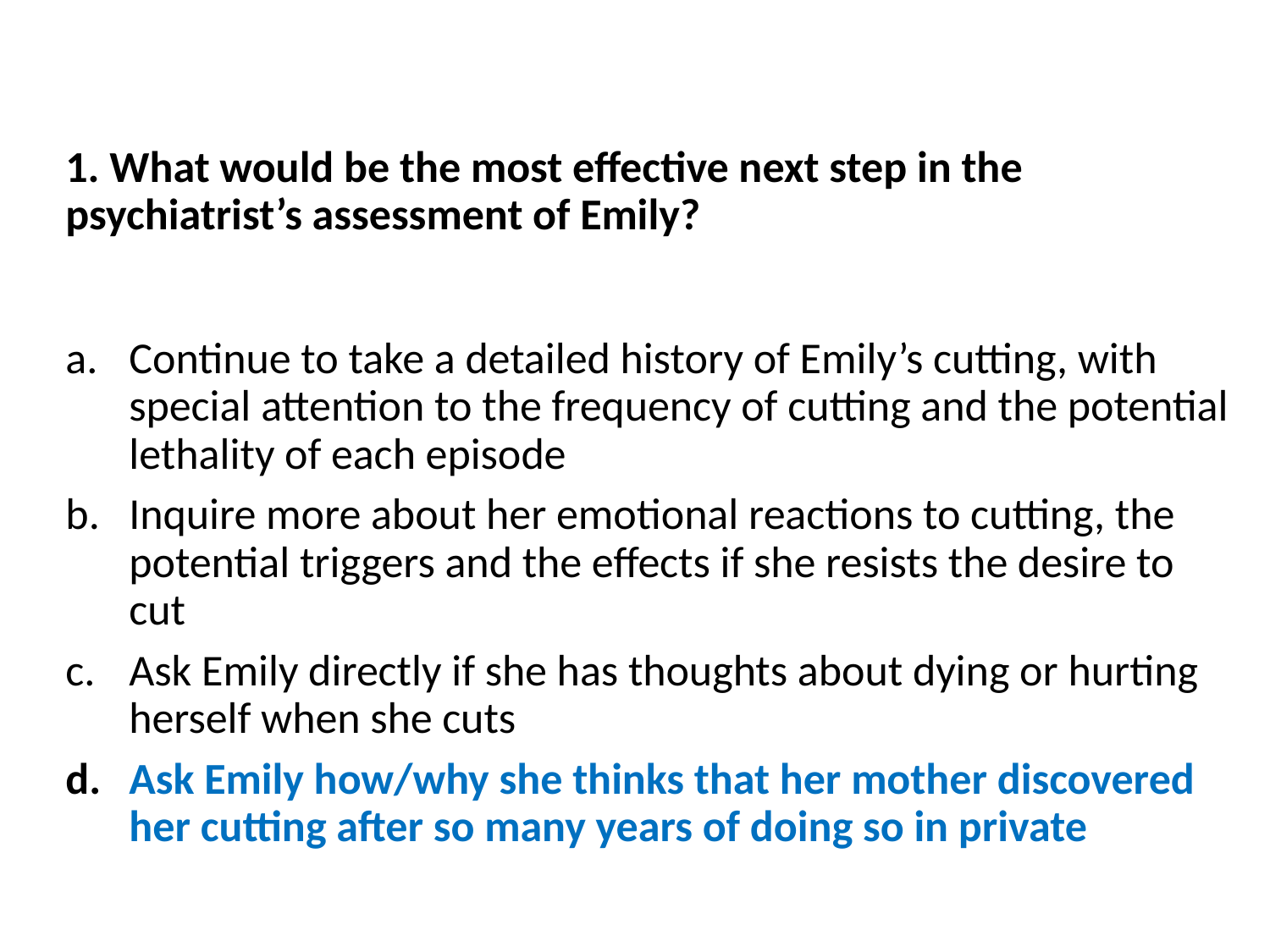

1. What would be the most effective next step in the psychiatrist’s assessment of Emily?
Continue to take a detailed history of Emily’s cutting, with special attention to the frequency of cutting and the potential lethality of each episode
Inquire more about her emotional reactions to cutting, the potential triggers and the effects if she resists the desire to cut
Ask Emily directly if she has thoughts about dying or hurting herself when she cuts
Ask Emily how/why she thinks that her mother discovered her cutting after so many years of doing so in private

## Slide 14
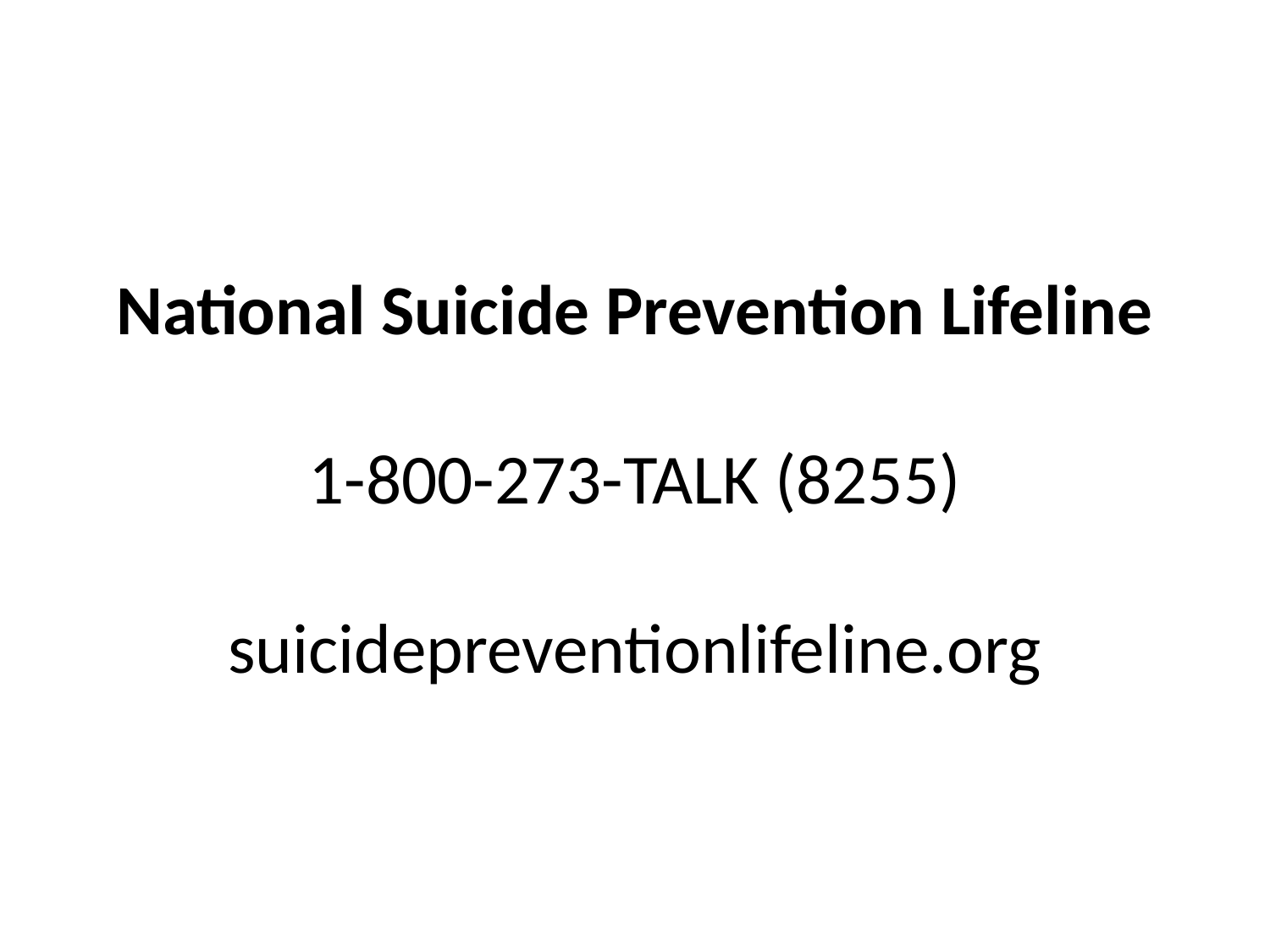

National Suicide Prevention Lifeline
1-800-273-TALK (8255)
suicidepreventionlifeline.org

## Slide 15
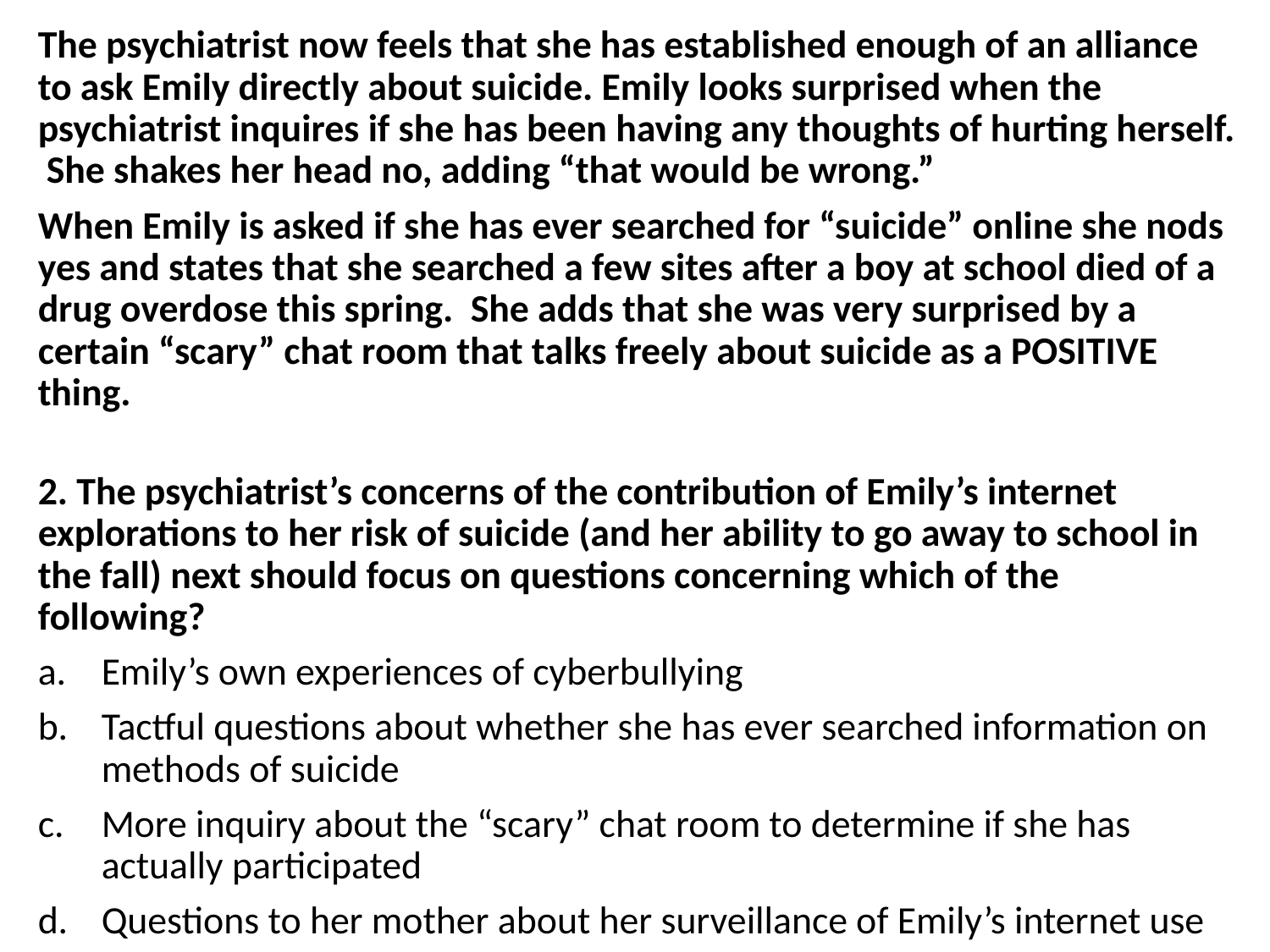

The psychiatrist now feels that she has established enough of an alliance to ask Emily directly about suicide. Emily looks surprised when the psychiatrist inquires if she has been having any thoughts of hurting herself. She shakes her head no, adding “that would be wrong.”
When Emily is asked if she has ever searched for “suicide” online she nods yes and states that she searched a few sites after a boy at school died of a drug overdose this spring. She adds that she was very surprised by a certain “scary” chat room that talks freely about suicide as a POSITIVE thing.
2. The psychiatrist’s concerns of the contribution of Emily’s internet explorations to her risk of suicide (and her ability to go away to school in the fall) next should focus on questions concerning which of the following?
Emily’s own experiences of cyberbullying
Tactful questions about whether she has ever searched information on methods of suicide
More inquiry about the “scary” chat room to determine if she has actually participated
Questions to her mother about her surveillance of Emily’s internet use

## Slide 16
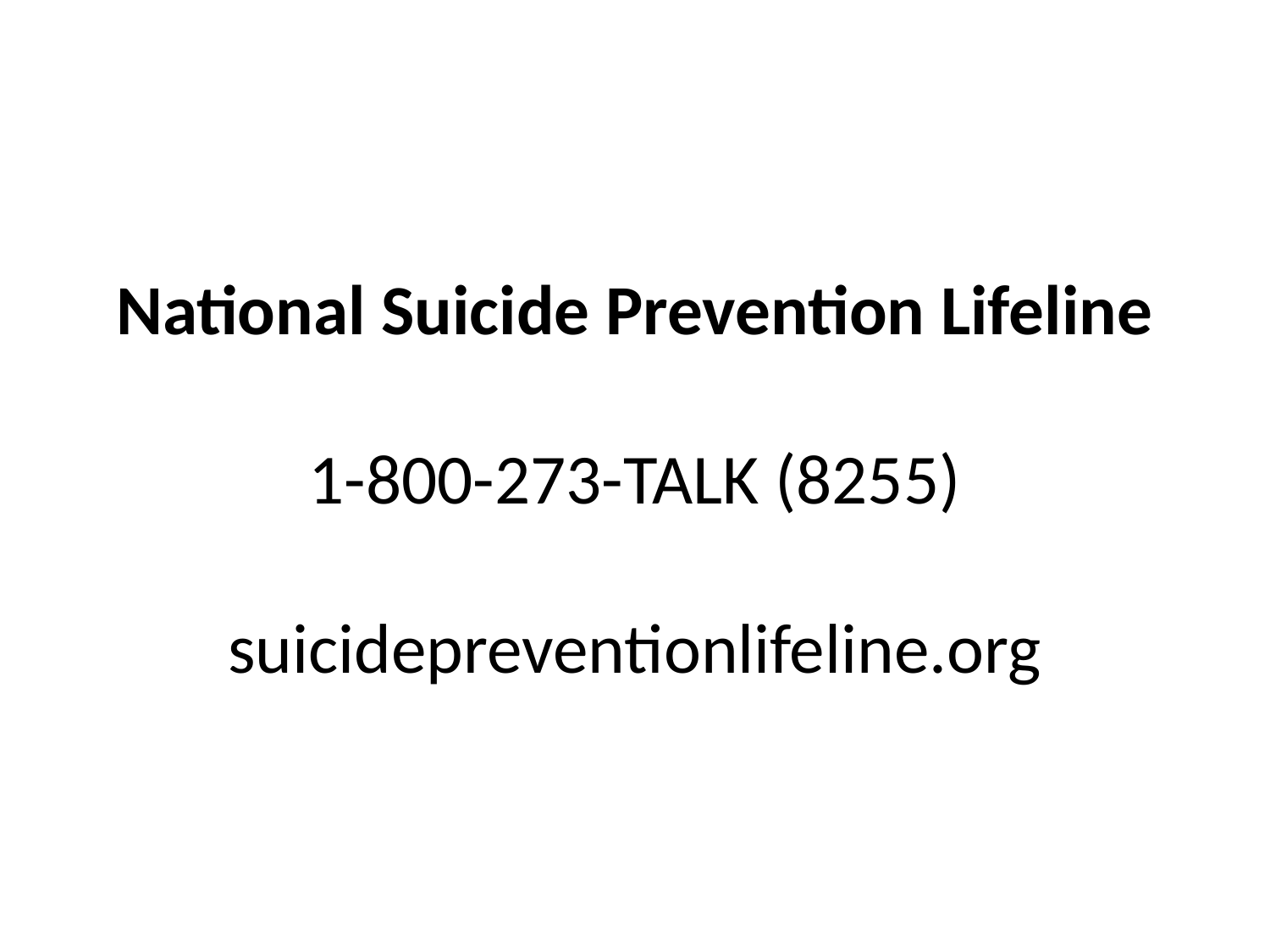

National Suicide Prevention Lifeline
1-800-273-TALK (8255)
suicidepreventionlifeline.org

## Slide 17
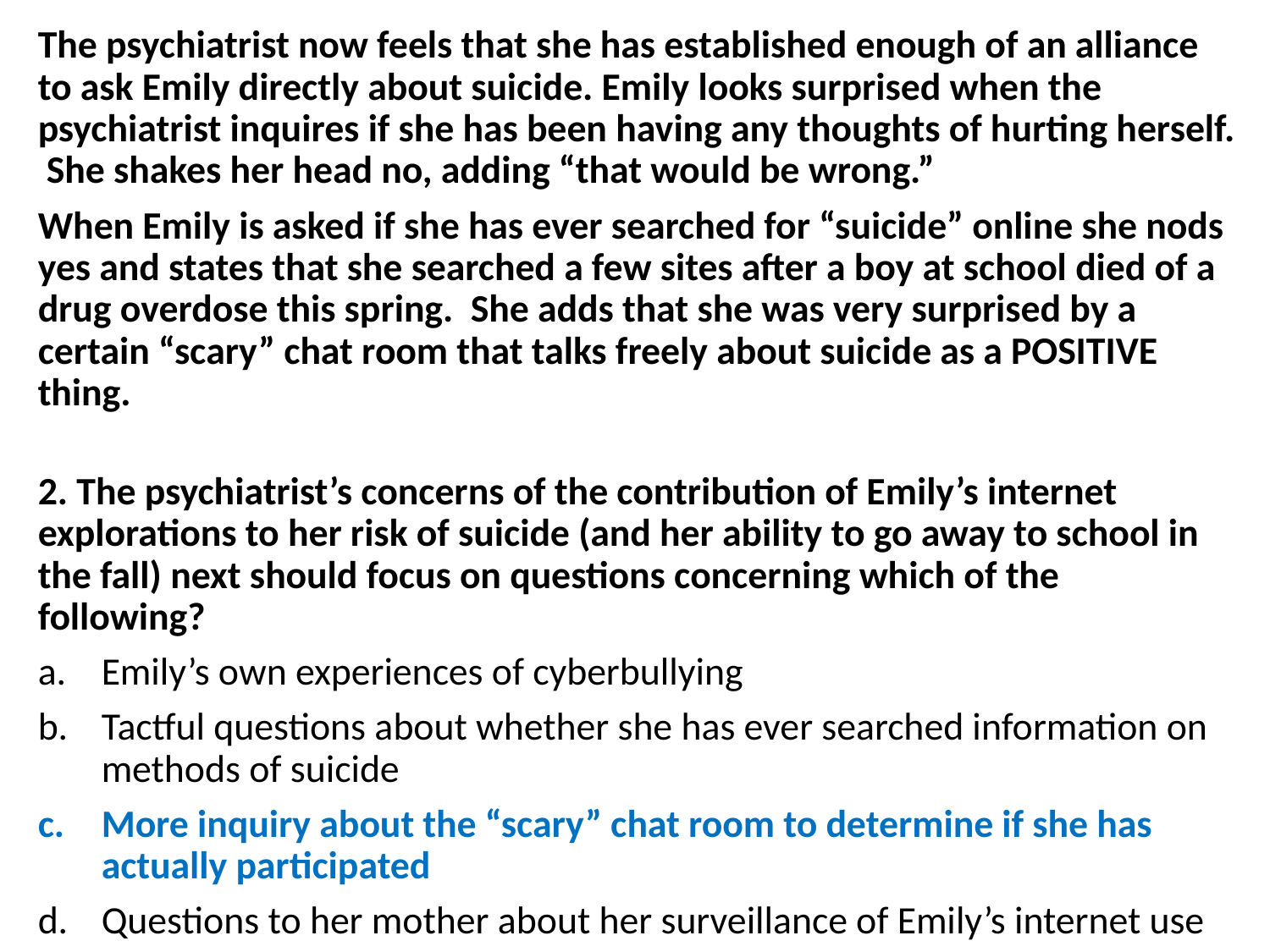

The psychiatrist now feels that she has established enough of an alliance to ask Emily directly about suicide. Emily looks surprised when the psychiatrist inquires if she has been having any thoughts of hurting herself. She shakes her head no, adding “that would be wrong.”
When Emily is asked if she has ever searched for “suicide” online she nods yes and states that she searched a few sites after a boy at school died of a drug overdose this spring. She adds that she was very surprised by a certain “scary” chat room that talks freely about suicide as a POSITIVE thing.
2. The psychiatrist’s concerns of the contribution of Emily’s internet explorations to her risk of suicide (and her ability to go away to school in the fall) next should focus on questions concerning which of the following?
Emily’s own experiences of cyberbullying
Tactful questions about whether she has ever searched information on methods of suicide
More inquiry about the “scary” chat room to determine if she has actually participated
Questions to her mother about her surveillance of Emily’s internet use

## Slide 18
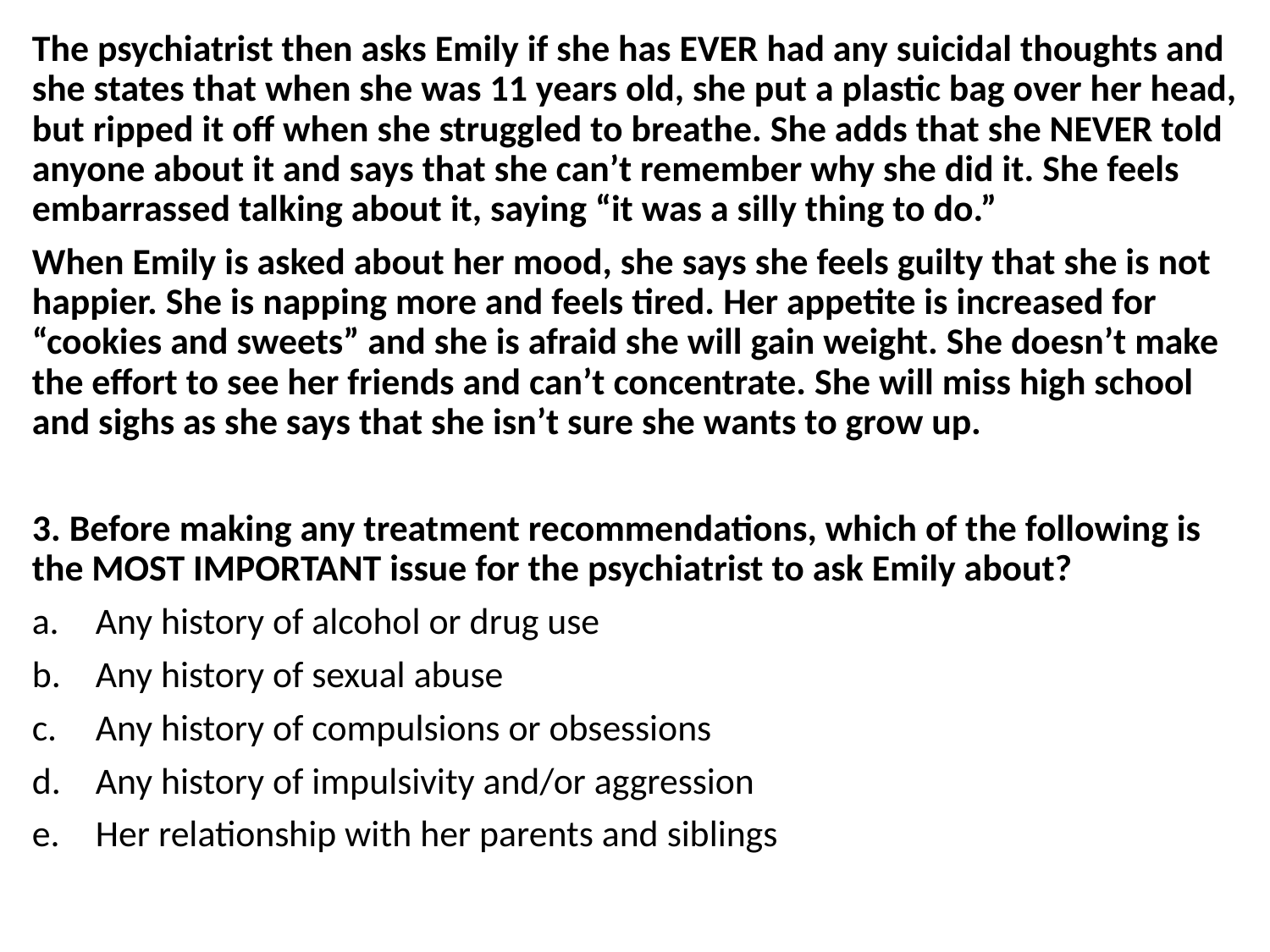

The psychiatrist then asks Emily if she has EVER had any suicidal thoughts and she states that when she was 11 years old, she put a plastic bag over her head, but ripped it off when she struggled to breathe. She adds that she NEVER told anyone about it and says that she can’t remember why she did it. She feels embarrassed talking about it, saying “it was a silly thing to do.”
When Emily is asked about her mood, she says she feels guilty that she is not happier. She is napping more and feels tired. Her appetite is increased for “cookies and sweets” and she is afraid she will gain weight. She doesn’t make the effort to see her friends and can’t concentrate. She will miss high school and sighs as she says that she isn’t sure she wants to grow up.
3. Before making any treatment recommendations, which of the following is the MOST IMPORTANT issue for the psychiatrist to ask Emily about?
Any history of alcohol or drug use
Any history of sexual abuse
Any history of compulsions or obsessions
Any history of impulsivity and/or aggression
Her relationship with her parents and siblings

## Slide 19
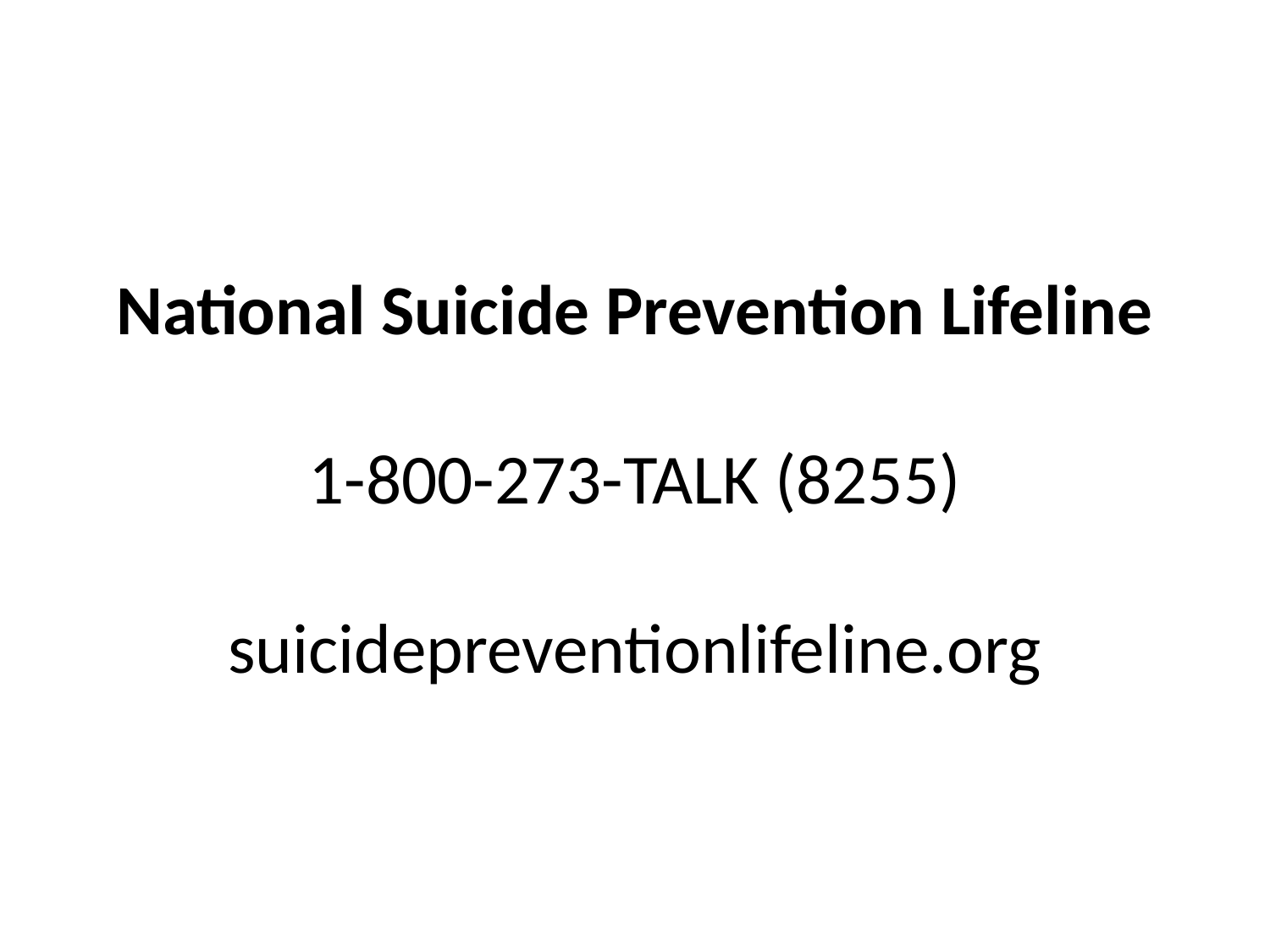

National Suicide Prevention Lifeline
1-800-273-TALK (8255)
suicidepreventionlifeline.org

## Slide 20
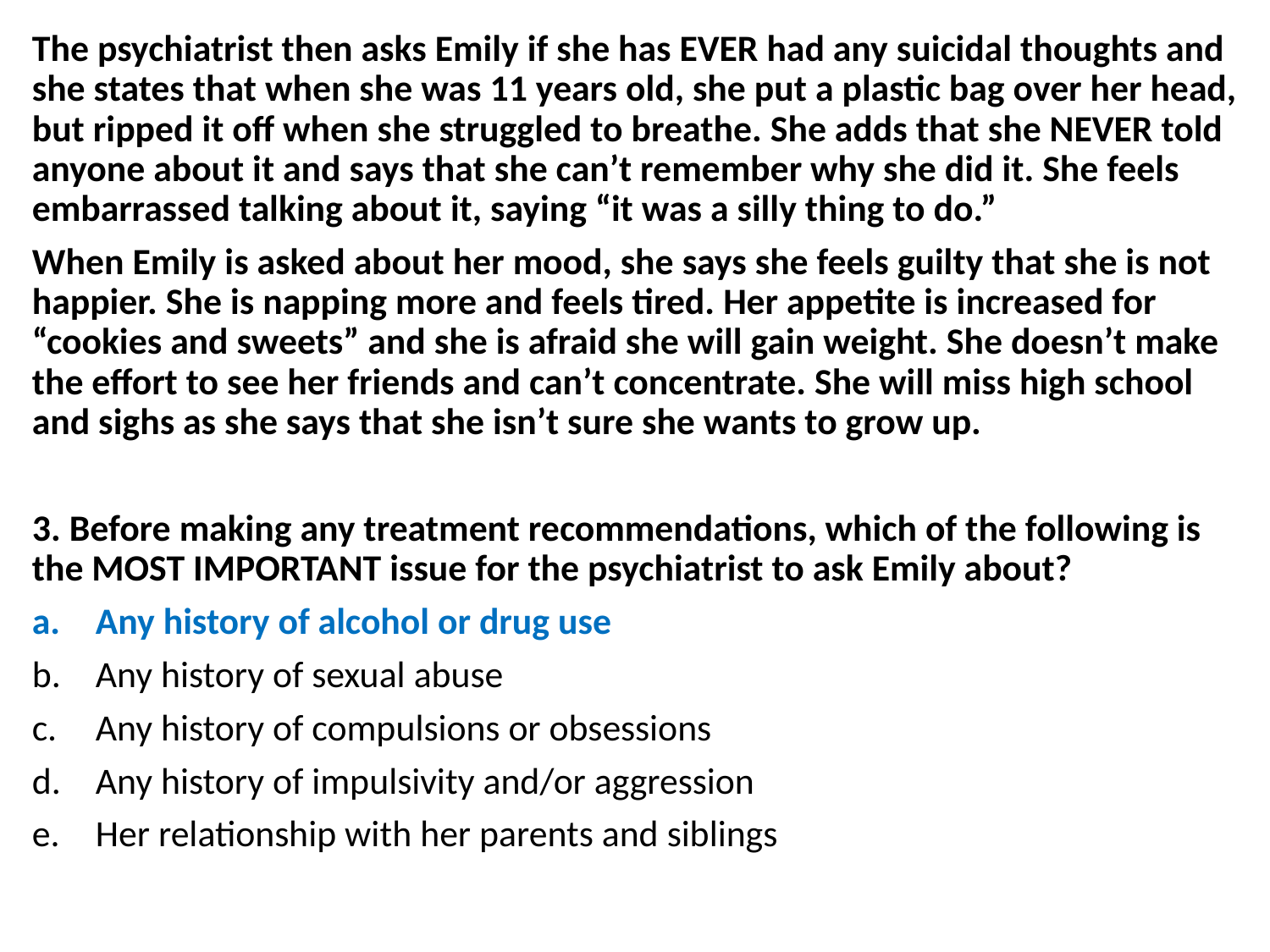

The psychiatrist then asks Emily if she has EVER had any suicidal thoughts and she states that when she was 11 years old, she put a plastic bag over her head, but ripped it off when she struggled to breathe. She adds that she NEVER told anyone about it and says that she can’t remember why she did it. She feels embarrassed talking about it, saying “it was a silly thing to do.”
When Emily is asked about her mood, she says she feels guilty that she is not happier. She is napping more and feels tired. Her appetite is increased for “cookies and sweets” and she is afraid she will gain weight. She doesn’t make the effort to see her friends and can’t concentrate. She will miss high school and sighs as she says that she isn’t sure she wants to grow up.
3. Before making any treatment recommendations, which of the following is the MOST IMPORTANT issue for the psychiatrist to ask Emily about?
Any history of alcohol or drug use
Any history of sexual abuse
Any history of compulsions or obsessions
Any history of impulsivity and/or aggression
Her relationship with her parents and siblings

## Slide 21
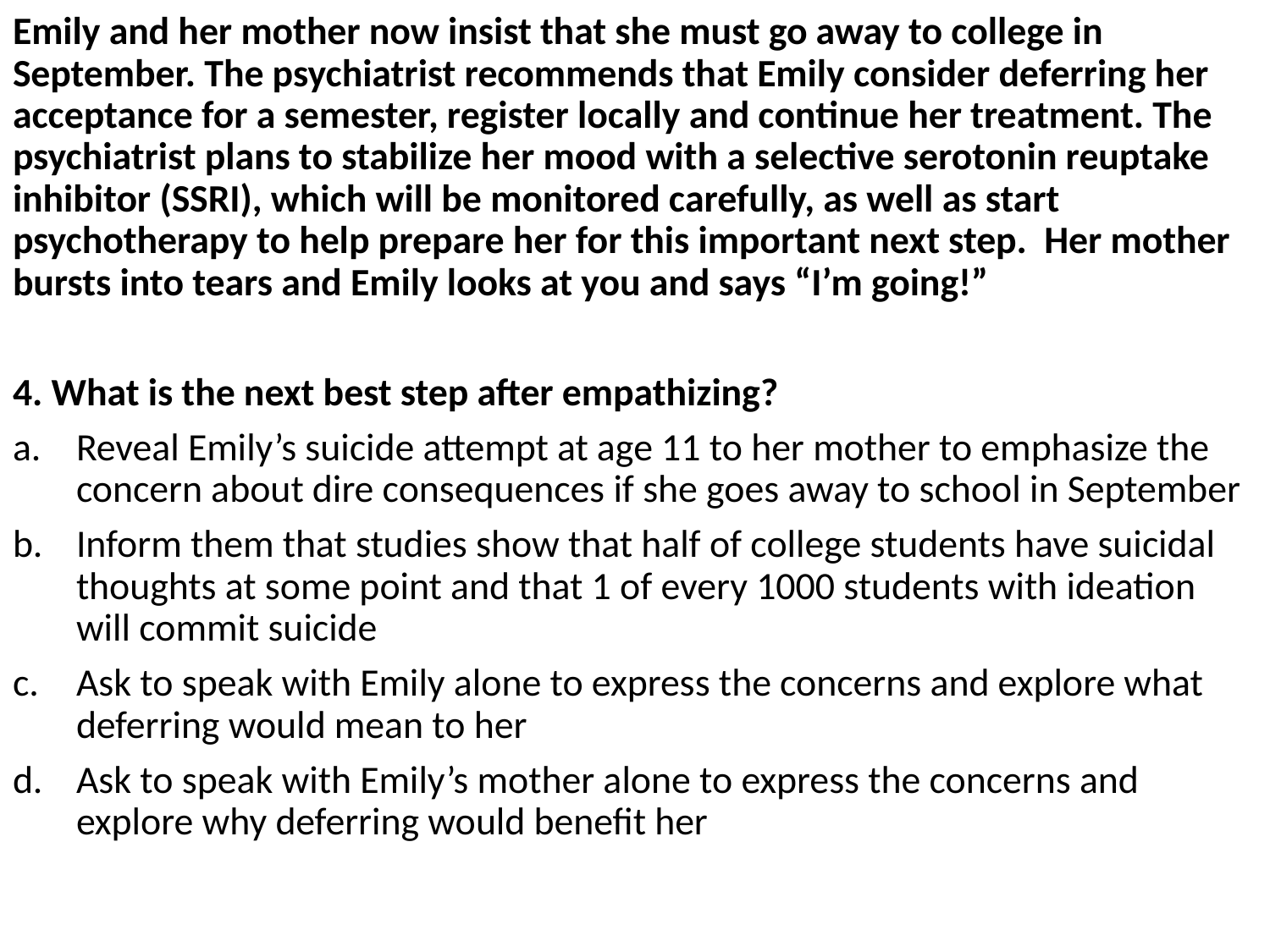

Emily and her mother now insist that she must go away to college in September. The psychiatrist recommends that Emily consider deferring her acceptance for a semester, register locally and continue her treatment. The psychiatrist plans to stabilize her mood with a selective serotonin reuptake inhibitor (SSRI), which will be monitored carefully, as well as start psychotherapy to help prepare her for this important next step. Her mother bursts into tears and Emily looks at you and says “I’m going!”
4. What is the next best step after empathizing?
Reveal Emily’s suicide attempt at age 11 to her mother to emphasize the concern about dire consequences if she goes away to school in September
Inform them that studies show that half of college students have suicidal thoughts at some point and that 1 of every 1000 students with ideation will commit suicide
Ask to speak with Emily alone to express the concerns and explore what deferring would mean to her
Ask to speak with Emily’s mother alone to express the concerns and explore why deferring would benefit her

## Slide 22
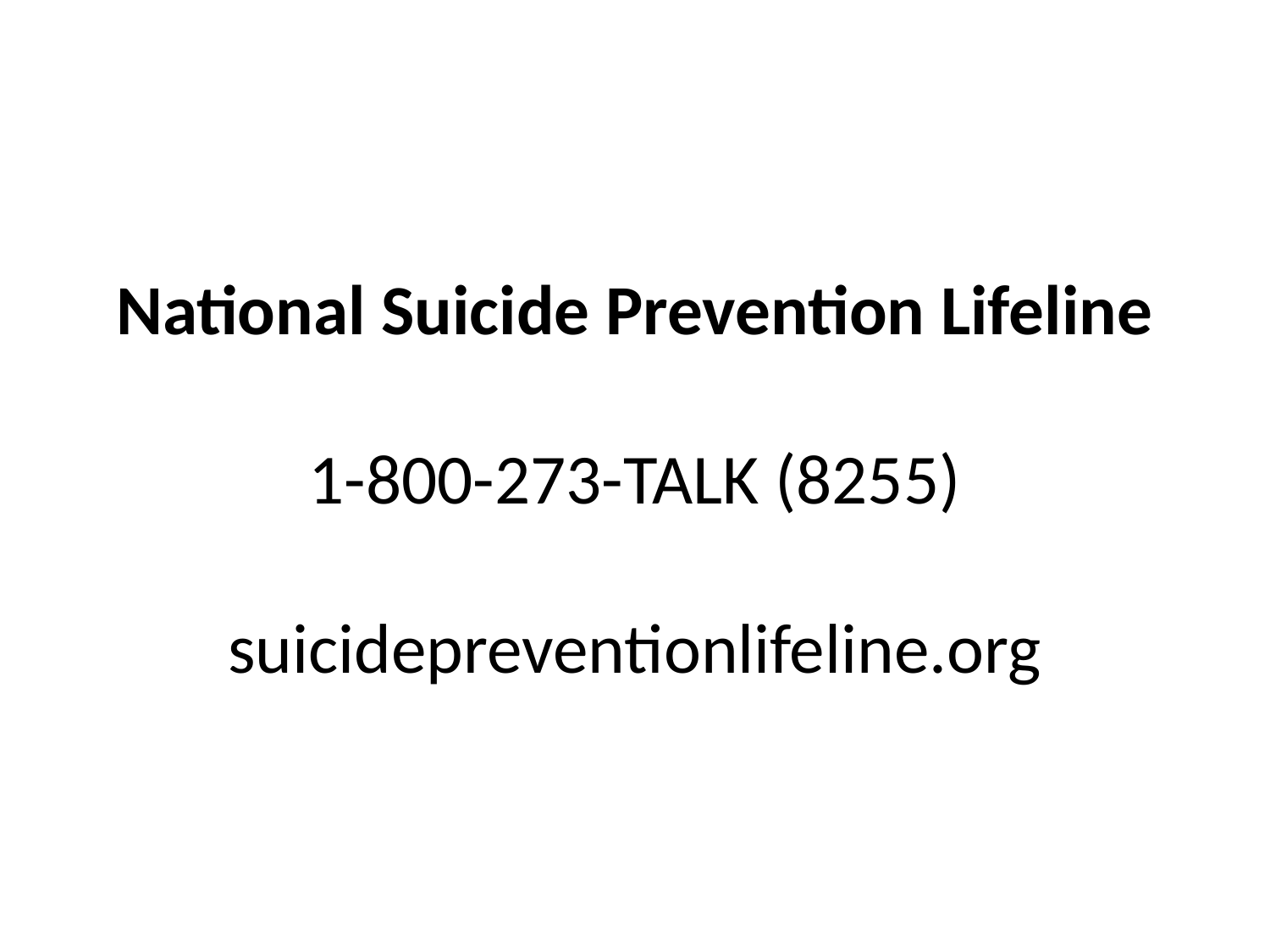

National Suicide Prevention Lifeline
1-800-273-TALK (8255)
suicidepreventionlifeline.org

## Slide 23
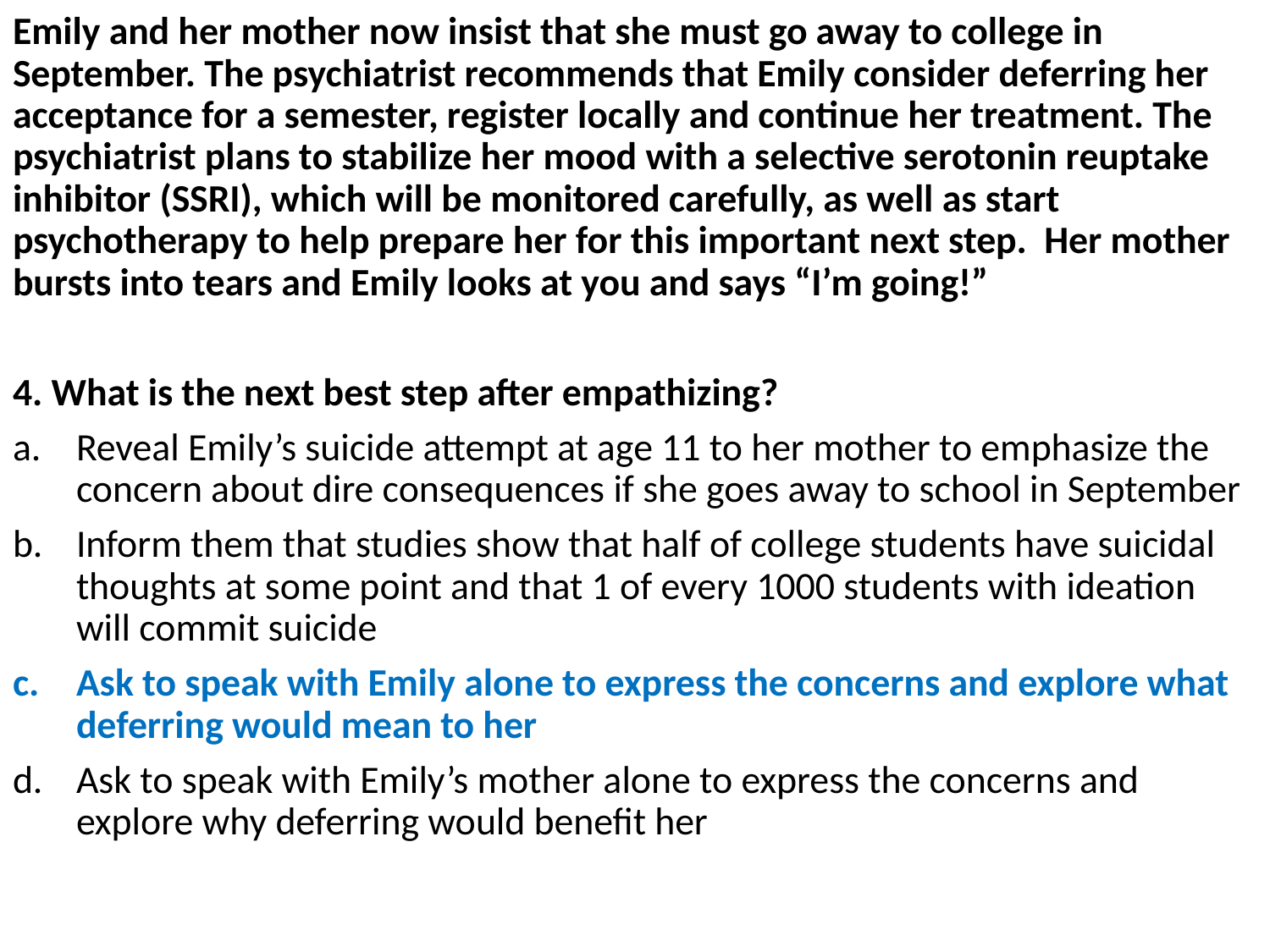

Emily and her mother now insist that she must go away to college in September. The psychiatrist recommends that Emily consider deferring her acceptance for a semester, register locally and continue her treatment. The psychiatrist plans to stabilize her mood with a selective serotonin reuptake inhibitor (SSRI), which will be monitored carefully, as well as start psychotherapy to help prepare her for this important next step. Her mother bursts into tears and Emily looks at you and says “I’m going!”
4. What is the next best step after empathizing?
Reveal Emily’s suicide attempt at age 11 to her mother to emphasize the concern about dire consequences if she goes away to school in September
Inform them that studies show that half of college students have suicidal thoughts at some point and that 1 of every 1000 students with ideation will commit suicide
Ask to speak with Emily alone to express the concerns and explore what deferring would mean to her
Ask to speak with Emily’s mother alone to express the concerns and explore why deferring would benefit her

## Slide 24
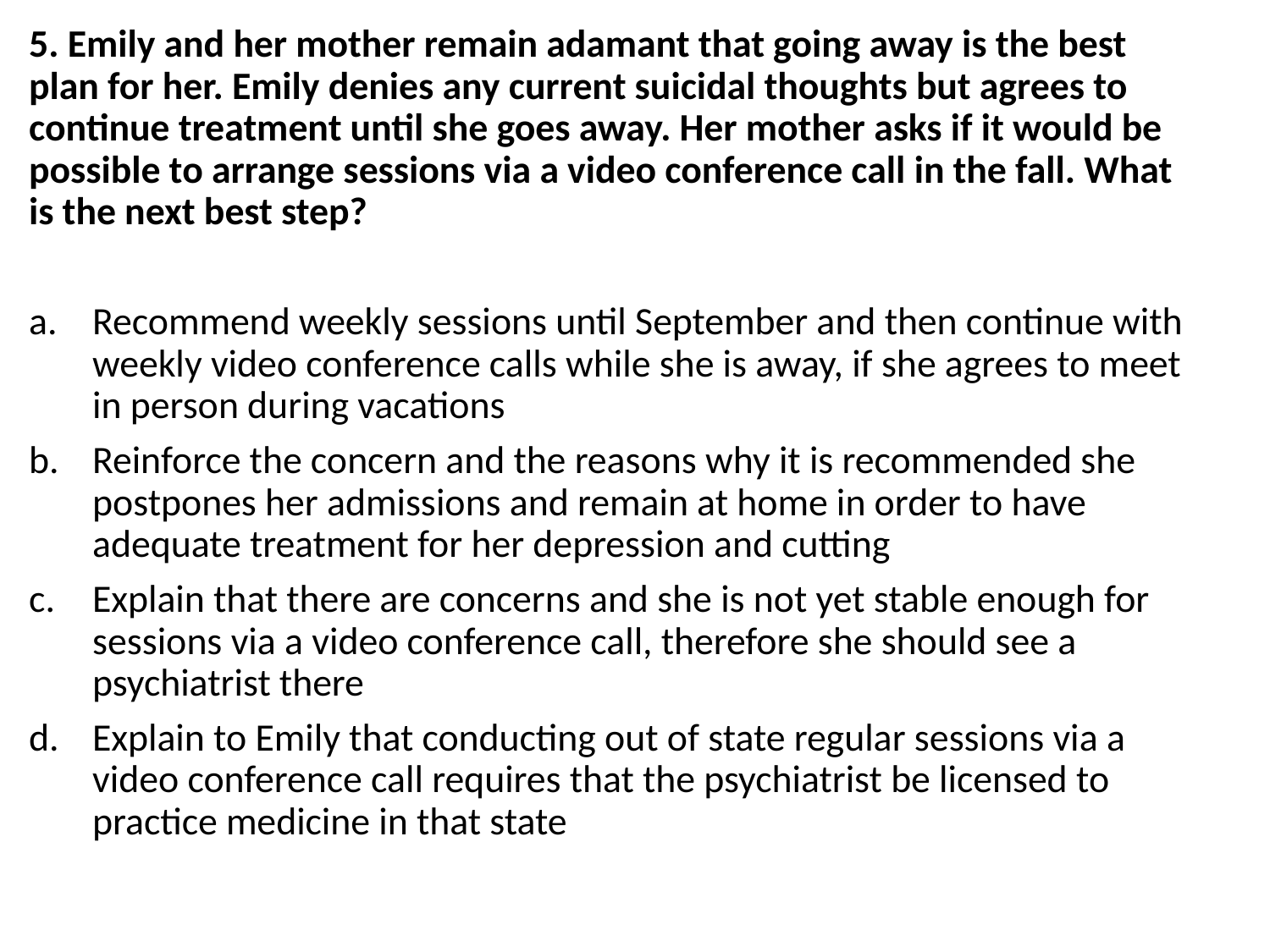

5. Emily and her mother remain adamant that going away is the best plan for her. Emily denies any current suicidal thoughts but agrees to continue treatment until she goes away. Her mother asks if it would be possible to arrange sessions via a video conference call in the fall. What is the next best step?
Recommend weekly sessions until September and then continue with weekly video conference calls while she is away, if she agrees to meet in person during vacations
Reinforce the concern and the reasons why it is recommended she postpones her admissions and remain at home in order to have adequate treatment for her depression and cutting
Explain that there are concerns and she is not yet stable enough for sessions via a video conference call, therefore she should see a psychiatrist there
Explain to Emily that conducting out of state regular sessions via a video conference call requires that the psychiatrist be licensed to practice medicine in that state

## Slide 25
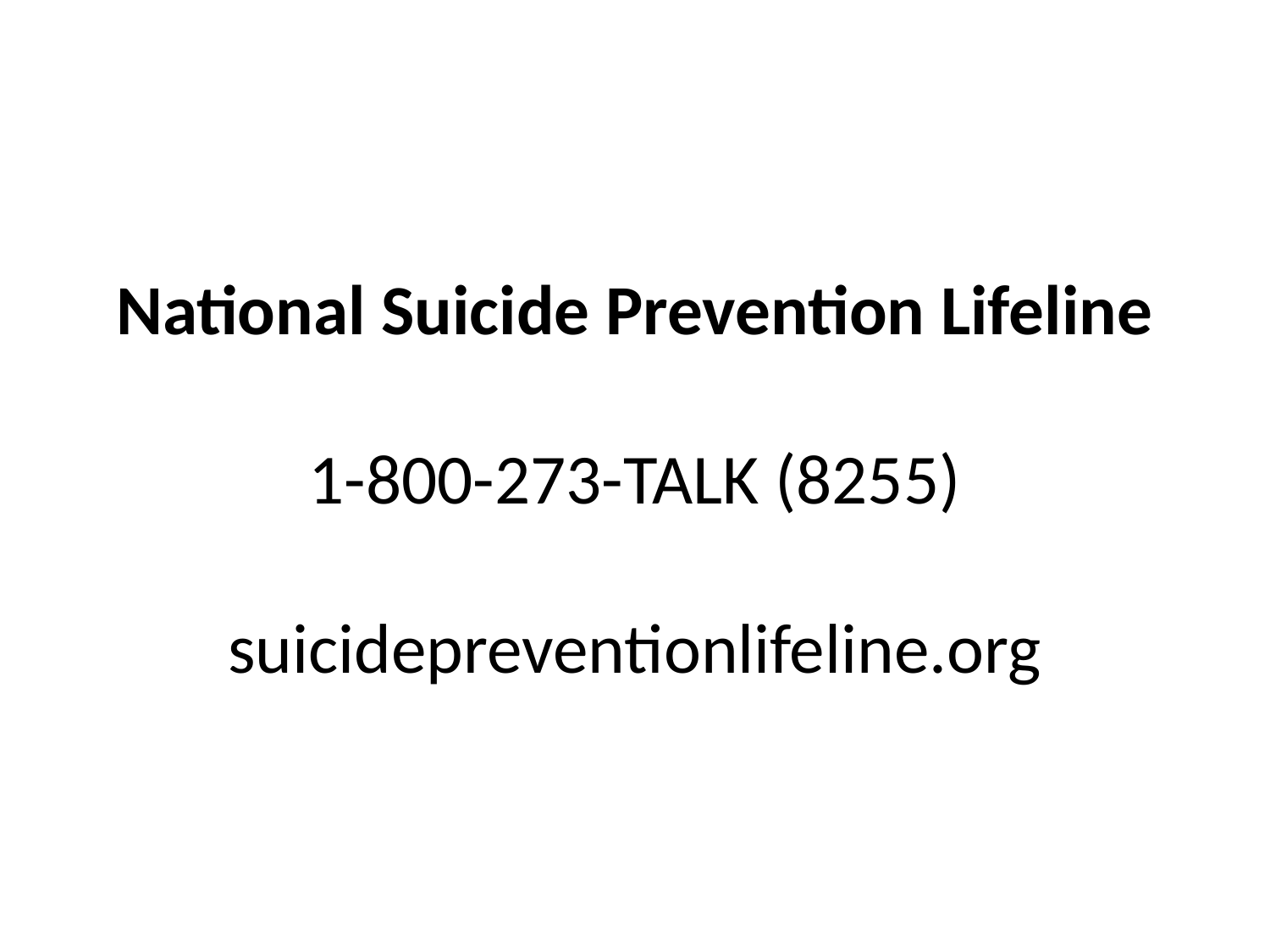

National Suicide Prevention Lifeline
1-800-273-TALK (8255)
suicidepreventionlifeline.org

## Slide 26
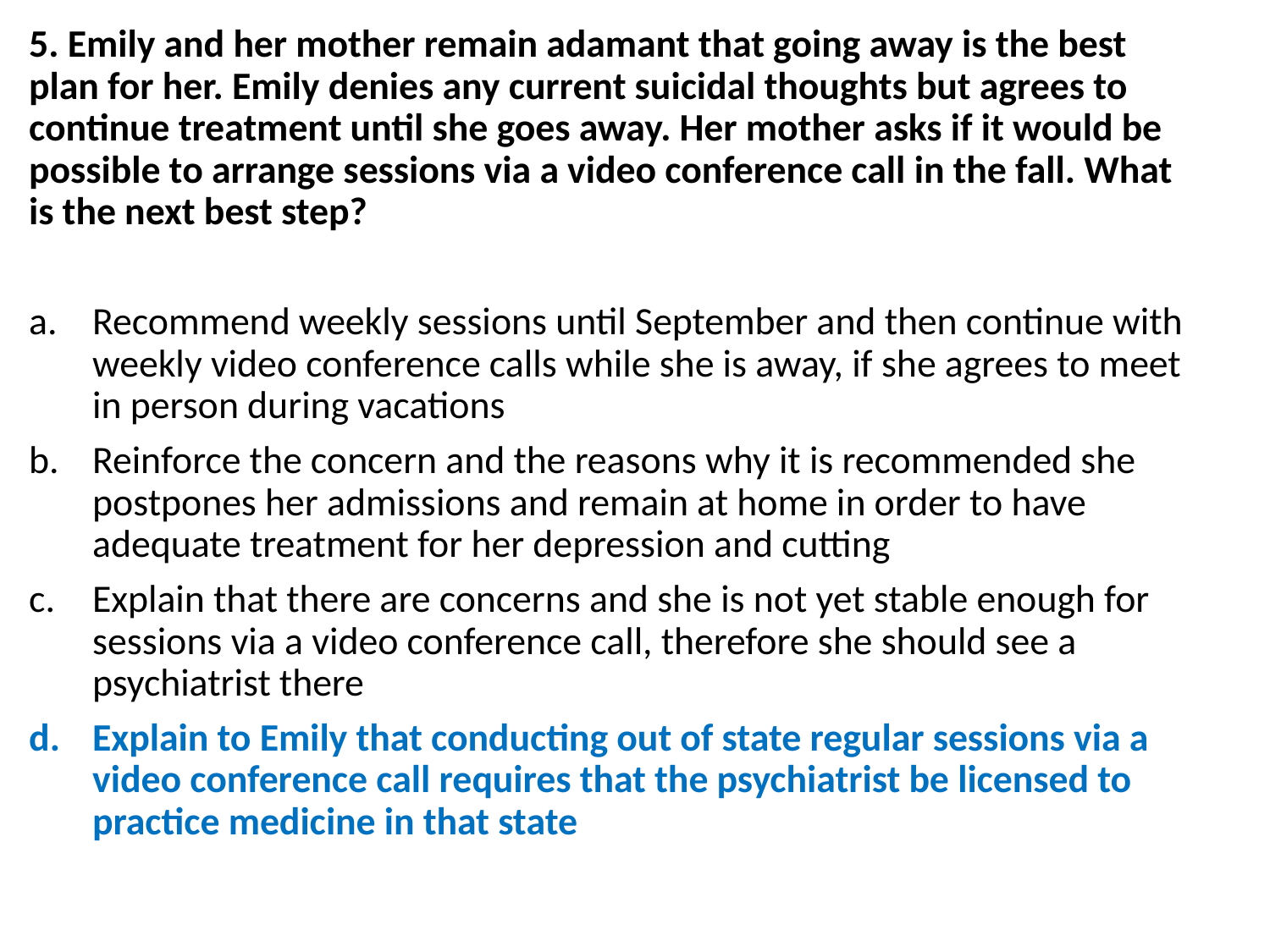

5. Emily and her mother remain adamant that going away is the best plan for her. Emily denies any current suicidal thoughts but agrees to continue treatment until she goes away. Her mother asks if it would be possible to arrange sessions via a video conference call in the fall. What is the next best step?
Recommend weekly sessions until September and then continue with weekly video conference calls while she is away, if she agrees to meet in person during vacations
Reinforce the concern and the reasons why it is recommended she postpones her admissions and remain at home in order to have adequate treatment for her depression and cutting
Explain that there are concerns and she is not yet stable enough for sessions via a video conference call, therefore she should see a psychiatrist there
Explain to Emily that conducting out of state regular sessions via a video conference call requires that the psychiatrist be licensed to practice medicine in that state

## Slide 27
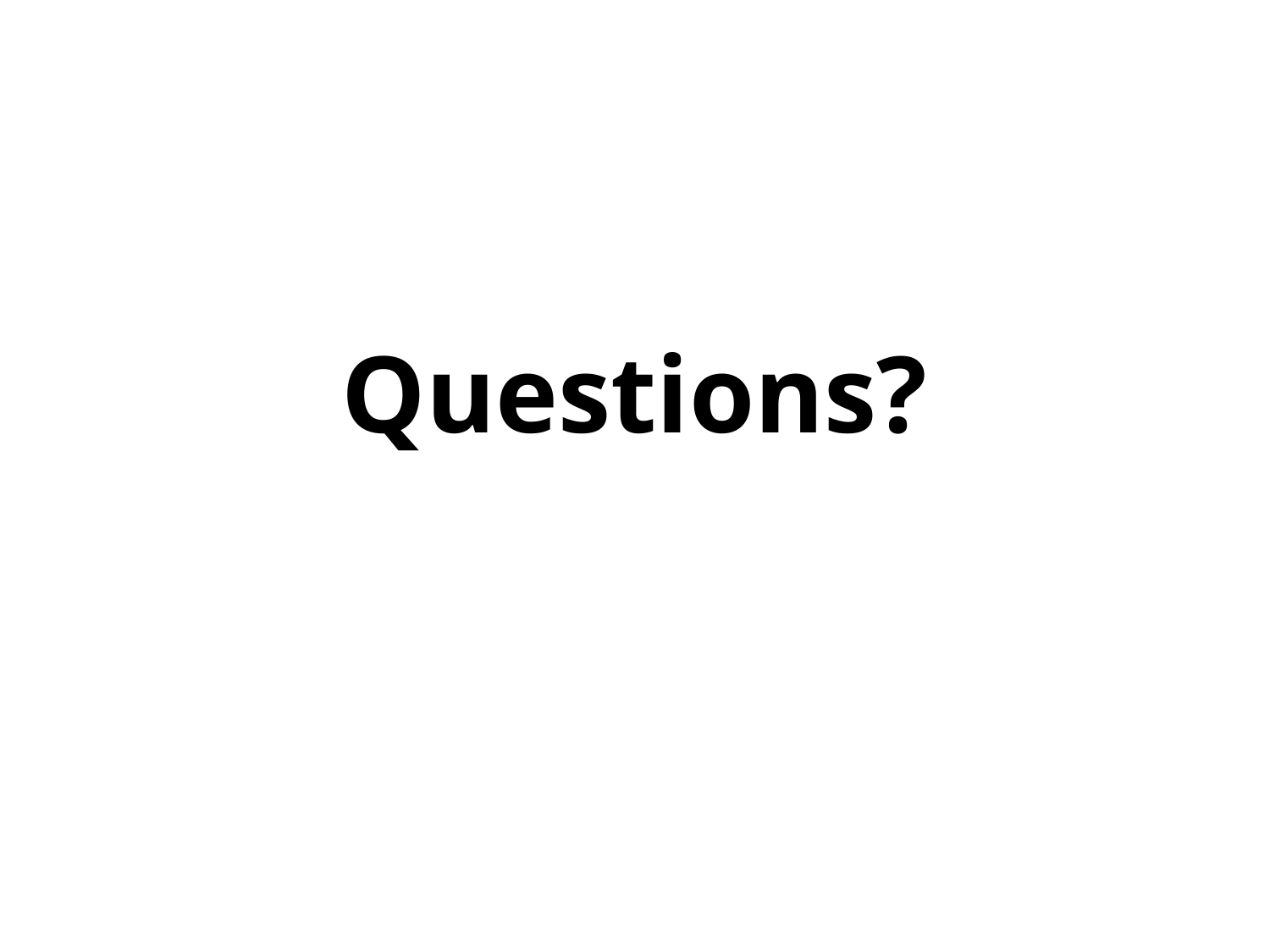

# Questions?
