# Supplementary material for: Suicide Assessment and Management Team-Based Learning Module
Source: MedEdPORTAL. 2020 Aug 20;16:10952. doi: 10.15766/mep_2374-8265.10952 (PMC7449577; doi:10.15766/mep_2374-8265.10952)
Supplement: Supplementary file 1 — Student Handout.docxReadiness Assurance Test Template.docxAppeal Form.docxPowerPoint Presentation Template.pptxReadiness Assurance Test Response Rates.docxApplication Exercise Response Rates.docxApplication Exercise Explanations.docx [file mep_2374-8265.10952-s001.zip › E. Readiness Assurance Test Response Rates.docx]

ATTENTION, STUDENTS: If you are accessing this material BEFORE it is used in your course, please do NOT read this document prior to the class session. An answer key is included in this module, which is designed to lead you through a learning experience that reinforces your knowledge of the content. Early review or dissemination of this material to others will diminish the learning opportunity and be considered academic misconduct.

**Suicide Assessment and Management TBL**

**Readiness Assurance Test (iRAT/tRAT)**

**Formatted to D014 IF-AT Card**

1. Suicide risk is reliably predicted by which *single* factor?

|  | **2016 iRAT Data** | **2017 iRAT Data** | **2018 iRAT Data** |
| --- | --- | --- | --- |
| - 1. Family history of mood disorders | 0/98 | 0/127 | 0/117 |
| - 1. Past suicide attempt | 42/98 | 52/127 | 50/117 |
| - 1. Persistent feelings of hopelessness | 0/98 | 1/127 | 0/117 |
| - 1. **None of the above** | 56/98 | 74/127 | 67/117 |
|  | **2016 tRAT:**  18/20 D;  2/20 B then D | **2017 tRAT:**  18/21 D;  3/21 B then D | **2018 tRAT:**  20/21 D;  1/21 B then D |

***Explanation:*** There is no pathognomonic risk factor for suicide. A single suicide risk factor does not have adequate statistical power on which to base assessment. Suicide risk assessment cannot be predicated on the basis of any one factor (Meltzer et al. 2003b); the assessment of suicide risk is multifactorial. (page 14)

****New mapping required if alternate preparatory assignment used***

1. What demographic has the *highest* suicide rate?

|  | **2016 iRAT Data** | **2017 iRAT Data** | **2018 iRAT Data** |
| --- | --- | --- | --- |
| - 1. African American males between 12-15 years of age | 0/98 | 0/127 | 0/117 |
| - 1. Hispanic females between 15-18 years of age | 1/98 | 2/127 | 0/117 |
| - 1. White females between 65-70 years of age | 1/98 | 2/127 | 2/117 |
| - 1. **White males between 85-90 years of age** | 96/98 | 123/127 | 115/117 |
|  | **2016 tRAT:** 20/20 D | **2017 tRAT:** 21/21 D | **2018 tRAT:** 21/21 D |

***Explanation:*** The suicide rates for white males 65 years and older are elevated. White males older than 85 have the highest suicide rates. Males die by suicide at a rate three to four times greater than that of females. Females make suicide attempts at a rate three to four times greater to that of men. Divorced individuals are at significantly increased risk compared to married individuals. The suicide rate is higher among white individuals (with the exception of young adults) than among African Americans. (page 16)

**New mapping required if alternate preparatory assignment used*

1. What is a *key difference* between suicidal adolescents and suicidal adults?

|  | **2016 iRAT Data** | **2017 iRAT Data** | **2018 iRAT Data** |
| --- | --- | --- | --- |
| - 1. Contagion effects are more powerful in suicidal adults. | 3/98 | 2/127 | 3/117 |
| - 1. **Serotonin reuptake inhibitors require more monitoring in suicidal adolescents.** | 76/98 | 92/127 | 80/117 |
| - 1. Suicide attempts are more common in suicidal adults. | 6/98 | 21/127 | 15/117 |
| - 1. Suicidal ideation is less likely to be denied when asked about in suicidal adolescents. | 13/98 | 12/127 | 19/117 |
|  | **2016 tRAT:** 20/20 B | **2017 tRAT:** 21/21 B | **2018 tRAT:**  19/21 B;  1/21 D then B;  1/21 C, D then B |

***Explanation:*** In the treatment of suicidal adolescents, selective serotonin reuptake inhibitors require more monitoring. In addition, family involvement in treatment is more important. See Table 20-1 for more information. (page 350)

**New mapping required if alternate preparatory assignment used*

1. Which antipsychotic medication is indicated for suicide risk reduction in patients with schizophrenia?

|  | **2016 iRAT Data** | **2017 iRAT Data** | **2018 iRAT Data** |
| --- | --- | --- | --- |
| - 1. **Clozapine** | 93/98 | 118/127 | 109/117 |
| - 1. Olanzapine | 0/98 | 1/127 | 1/117 |
| - 1. Risperidone | 4/98 | 5/127 | 7/117 |
| - 1. Thioridazine | 1/98 | 3/127 | 0/117 |
|  | **2016 tRAT:** 20/20 D | **2017 tRAT:** 21/21 A | **2018 tRAT:** 21/21 A |

***Explanation:*** The most data on risk reduction of suicidal behaviors exist for the atypical antipsychotic clozapine, the only treatment approved by the FDA for suicide risk reduction, although the indication is limited to patients with schizophrenia. (page 224)

**New mapping required if alternate preparatory assignment used*

1. Suicide is the leading cause of death among persons younger than 35 years of age with what psychiatric diagnosis?

|  | **2016 iRAT Data** | **2017 iRAT Data** | **2018 iRAT Data** |
| --- | --- | --- | --- |
| 1. Attention deficit hyperactivity disorder | 0/98 | 0/127 | 0/117 |
| 1. Bipolar disorder | 2/98 | 9/127 | 12/117 |
| 1. Depression | 10/98 | 15/127 | 7/117 |
| 1. **Schizophrenia** | 86/98 | 103/127 | 98/117 |
|  | **2016 tRAT:** 20/20 D | **2017 tRAT:** 21/21 D | **2018 tRAT:** 21/21 D |

***Explanation:*** Suicide is the leading cause of death among persons with schizophrenia who are younger than 35 years of age. (page 225)

**New mapping required if alternate preparatory assignment used*

NEW Questions that May be Used for the Readiness Assurance Test

1) A 32-year-old woman presents to her Psychiatrist for management of major depressive disorder. She started an antidepressant the week prior, but still feels hopeless. At the appointment, the Psychiatrist notes that the patient is agitated. Her past medical history is also significant for panic attacks, type 2 diabetes, and hypothyoridism. Which of the following medications should be included in her treatment regimen while the antidepressant is being given the opportunity to work?

- 1. Clonidine
  2. Gabapentin
  3. **Lorazepam**
  4. Mirtazapine
  5. Quetiapine

Explanation: The combination of severe depression and anxiety or panic attacks can prove lethal. A patient may be able to tolerate depression. When anxiety or panic is also present, the patient’s life may become unbearable, and suicide risk is dangerously elevated. Anxiety (agitation) symptoms should be treated aggressively while antidepressant medications are being given an opportunity to work. (page 223)

2) A 16-year-old presents to his pediatrician for his annual physical. At the end of the appointment, the boy mentions that he is struggling with bullying at school and has recently experienced suicidal thoughts. What must be immediately included in the pediatrician’s assessment, evaluation, and plan for this patient?

- 1. **Ask about presence of firearms at home**
  2. Advise anti-bullying campaign at school
  3. Initiate antidepressant therapy
  4. Invite parents to appointment for discussion

Explanation: Although sex differences are explained in part by the means employed— teenage males tend to use more lethal methods, such as firearms and hanging, rather than the less dangerous methods often used by females, such as poisoning. (page 350)
