# Supplementary material for: Suicide Assessment and Management Team-Based Learning Module
Source: MedEdPORTAL. 2020 Aug 20;16:10952. doi: 10.15766/mep_2374-8265.10952 (PMC7449577; doi:10.15766/mep_2374-8265.10952)
Supplement: Supplementary file 1 — Student Handout.docxReadiness Assurance Test Template.docxAppeal Form.docxPowerPoint Presentation Template.pptxReadiness Assurance Test Response Rates.docxApplication Exercise Response Rates.docxApplication Exercise Explanations.docx [file mep_2374-8265.10952-s001.zip › F. Application Exercise Response Rates.docx]

**Suicide Assessment and Management TBL**

**Application Exercise Response Rates**

ATTENTION, STUDENTS: If you are accessing this material BEFORE it is used in your course, please do NOT read this document prior to the class session. An answer key is included in this module, which is designed to lead you through a learning experience that reinforces your knowledge of the content. Early review or dissemination of this material to others will diminish the learning opportunity and be considered academic misconduct.

****** Application questions were modified after the first year of implementation, therefore two years of response rates are provided (versus the three years included for the readiness assurance questions) ******

**Case**

Emily is a 17-year-old student brought to a psychiatrist’s office by her mother who recently discovered that she is cutting herself on the wrist and thigh. It is August and Emily is planning to attend an Ivy League university in September. Her mother is worried: Emily has worked so hard. Is it safe for her to go to college? Her mother reports that Emily is a shy girl. She has a few good friends (who are younger) and has never had a boyfriend. She is repeatedly described as “a good girl” who is nice to her siblings and has never been in trouble. The mother says Emily is more withdrawn this summer, sleeping more and spending hours online. She works in the local library 25 hours per week.

Emily reluctantly tells you that she started cutting when she was twelve because “it makes me feel better.” She admits to cutting more often in the past few months and keeps a hidden supply of razor blades. She never cuts deeply and is careful to avoid areas that might be visible.

**Question 1**

What would be the most effective next step in the psychiatrist’s assessment of Emily?

|  | **2017** | **2018** |
| --- | --- | --- |
| A. Continue to take a detailed history of Emily’s cutting, with special attention to the frequency of cutting and the potential lethality of each episode | 6/21 | 2/21 |
| B. Inquire more about her emotional reactions to cutting, the potential triggers and the effects if she resists the desire to cut | 15/21 | 13/21 |
| C. Ask Emily directly if she has thoughts about dying or hurting herself when she cuts | 0/21 | 4/21 |
| **D. Ask Emily how/why she thinks that her mother discovered her cutting after so many years of doing so in private** | 0/21 | 0/21 |
|  |  | **Two teams did not respond* |

**Question 2**

The psychiatrist now feels that she has established enough of an alliance to ask Emily directly about suicide. Emily looks surprised when the psychiatrist inquires if she has been having any thoughts of hurting herself. She shakes her head no, adding “that would be wrong.”

When Emily is asked if she has ever searched for “suicide” online she nods yes and states that she searched a few sites after a boy at school died of a drug overdose this spring. She adds that she was very surprised by a certain “scary” chat room that talks freely about suicide as a POSITIVE thing.

The psychiatrist’s concerns of the contribution of Emily’s internet explorations to her risk of suicide (and her ability to go away to school in the fall) next should focus on questions concerning which of the following?

|  | **2017** | **2018** |
| --- | --- | --- |
| A. Emily’s own experiences of cyberbullying | 0/21 | 0/21 |
| B**.** Tactful questions about whether she has ever searched information on methods of suicide | 6/21 | 6/21 |
| C. **More inquiry about the “scary” chat room to determine if she has actually participated** | 11/21 | 14/21 |
| D. Questions to her mother about her surveillance of Emily’s internet use | 0/21 | 0/21 |
|  | **Four teams did not respond* | **One team did not respond* |

**Question 3**

The psychiatrist then asks Emily if she has EVER had any suicidal thoughts and she states that when she was 11 years old, she put a plastic bag over her head, but ripped it off when she struggled to breathe. She adds that she NEVER told anyone about it and says that she can’t remember why she did it. She feels embarrassed talking about it, saying “it was a silly thing to do.”

When Emily is asked about her mood, she says she feels guilty that she is not happier. She is napping more and feels tired. Her appetite is increased for “cookies and sweets” and she is afraid she will gain weight. She doesn’t make the effort to see her friends and can’t concentrate. She will miss high school and sighs as she says that she isn’t sure she wants to grow up.

Before making any treatment recommendations, which of the following is the MOST IMPORTANT issue for the psychiatrist to ask Emily about?

|  | **2017** | **2018** |
| --- | --- | --- |
| **A. Any history of alcohol or drug use** | 4/21 | 0/21 |
| B. Any history of sexual abuse | 0/21 | 1/21 |
| C. Any history of compulsions or obsessions | 0/21 | 0/21 |
| D. Any history of impulsivity and/or aggression | 11/21 | 8/21 |
| E. Her relationship with her parents and siblings | 6/21 | 11/21 |
|  |  | **One team did not respond* |

**Question 4**

Emily and her mother now insist that she must go away to college in September. The psychiatrist recommends that Emily consider deferring her acceptance for a semester, register locally and continue her treatment. The psychiatrist plans to stabilize her mood with a selective serotonin reuptake inhibitor (SSRI), which will be monitored carefully, as well as start psychotherapy to help prepare her for this important next step. Her mother bursts into tears and Emily looks at you and says “I’m going!”

What is the next best step after empathizing?

|  | **2017** | **2018** |
| --- | --- | --- |
| A. Reveal Emily’s suicide attempt at age 11 to her mother to emphasize the concern about dire consequences if she goes away to school in September | 0/21 | 0/21 |
| B. Inform them that studies show that half of college students have suicidal thoughts at some point and that 1 of every 1000 students with ideation will commit suicide | 0/21 | 0/21 |
| C. **Ask to speak with Emily alone to express the concerns and explore what deferring would mean to her** | 21/21 | 21/21 |
| D. Ask to speak with Emily’s mother alone to express the concerns and explore why deferring would benefit her | 0/21 | 0/21 |

**Question 5**

Emily and her mother remain adamant that going away is the best plan for her. Emily denies any current suicidal thoughts but agrees to continue treatment until she goes away. Her mother asks if it would be possible to arrange sessions via a video conference call in the fall.

What is the next best step?

|  | **2017** | **2018** |
| --- | --- | --- |
| A Recommend weekly sessions until September and then continue with weekly video conference calls while she is away, if she agrees to meet in person during vacations | *Did not complete TBL module in allotted time frame | 5/21 |
| B. Reinforce the concern and the reasons why it is recommended she postpones her admissions and remain at home in order to have adequate treatment for her depression and cutting |  | 2/21 |
| C. Explain that there are concerns and she is not yet stable enough for sessions via a video conference call, therefore she should see a psychiatrist there |  | 9/21 |
| **D. Explain to Emily that conducting out of state regular sessions via a video conference call requires that the psychiatrist be licensed to practice medicine in that state** |  | 3/21 |
|  |  | **Two teams did not respond* |
