# Supplementary material for: Suicide Assessment and Management Team-Based Learning Module
Source: MedEdPORTAL. 2020 Aug 20;16:10952. doi: 10.15766/mep_2374-8265.10952 (PMC7449577; doi:10.15766/mep_2374-8265.10952)
Supplement: Supplementary file 1 — Student Handout.docxReadiness Assurance Test Template.docxAppeal Form.docxPowerPoint Presentation Template.pptxReadiness Assurance Test Response Rates.docxApplication Exercise Response Rates.docxApplication Exercise Explanations.docx [file mep_2374-8265.10952-s001.zip › G. Application Exercise Explanations.docx]

**Suicide Assessment and Management TBL**

**Application Exercise Response Rates**

ATTENTION, STUDENTS: If you are accessing this material BEFORE it is used in your course, please do NOT read this document prior to the class session. An answer key is included in this module, which is designed to lead you through a learning experience that reinforces your knowledge of the content. Early review or dissemination of this material to others will diminish the learning opportunity and be considered academic misconduct.

****** In 2015, a different case was used. Due to copyright laws, it cannot be published. For this reason, the application questions have two years of response rates (versus the three years included for the readiness assurance questions) ******

**Case**

Emily is a 17-year-old student brought to a psychiatrist’s office by her mother who recently discovered that she is cutting herself on the wrist and thigh. It is August and Emily is planning to attend an Ivy League university in September. Her mother is worried: Emily has worked so hard. Is it safe for her to go to college? Her mother reports that Emily is a shy girl. She has a few good friends (who are younger) and has never had a boyfriend. She is repeatedly described as “a good girl” who is nice to her siblings and has never been in trouble. The mother says Emily is more withdrawn this summer, sleeping more and spending hours online. She works in the local library 25 hours per week.

Emily reluctantly tells you that she started cutting when she was twelve because “it makes me feel better.” She admits to cutting more often in the past few months and keeps a hidden supply of razor blades. She never cuts deeply and is careful to avoid areas that might be visible.

**Question 1**

| What would be the most effective next step in the psychiatrist’s assessment of Emily?   1. Continue to take a detailed history of Emily’s cutting, with special attention to the frequency of cutting and the potential lethality of each episode 2. Inquire more about her emotional reactions to cutting, the potential triggers and the effects if she resists the desire to cut 3. Ask Emily directly if she has thoughts about dying or hurting herself when she cuts 4. **Ask Emily how/why she thinks that her mother discovered her cutting after so many years of doing so in private** |
| --- |

**Explanation:** This exercise is designed to introduce students to the important clinical consideration of the ‘why now’ question in the psychiatric interview, especially during the evaluation of suicide risk. It begins with the clinical assessment of Emily’s understanding of her current situation (i.e. Does she have any idea why the cutting has been discovered now after so many years? Does she have any awareness of her current feelings that have caused her to withdraw?) To move forward too quickly into a detailed inquiry about the cutting, or to transition abruptly to direct suicide risk assessment questions prior to establishing a rapport, could undermine Emily’s willingness to share sensitive information.

**Potential Challenges for Students:** All of these questions are essential to the risk assessment, especially the question about her emotional reactions to the cutting and awareness of triggers, but the order of inquiry will shape Emily’s comfort level with answering truthfully.

**Opportunities for Further Discussion:** Further opportunities to expand the conversation may include:

- Discussion of the role of psychiatric diagnoses as major risk factors for suicide
  - Emily is demonstrating symptoms of depression and has a long history of cutting (which increases risk of suicide). Students may discuss ruling out major depressive disorder and may consider bipolar depression or prodromal schizophrenia.
- Discussion of Emily’s passivity, lack of age-appropriate social life, and hints of immaturity that may make going away to college problematic and increase her suicide risk.

**Case – Part 2**

| The psychiatrist now feels that she has established enough of an alliance to ask Emily directly about suicide. Emily looks surprised when the psychiatrist inquires if she has been having any thoughts of hurting herself. She shakes her head no, adding “that would be wrong.”  When Emily is asked if she has ever searched for “suicide” online she nods yes and states that she searched a few sites after a boy at school died of a drug overdose this spring. She adds that she was very surprised by a certain “scary” chat room that talks freely about suicide as a POSITIVE thing.  **Question 2**  The psychiatrist’s concerns of the contribution of Emily’s internet explorations to her risk of suicide (and her ability to go away to school in the fall) next should focus on questions concerning which of the following?   1. Emily’s own experiences of cyberbullying 2. Tactful questions about whether she has ever searched information on methods of suicide 3. **More inquiry about the “scary” chat room to determine if she has actually participated** 4. Questions to her mother about her surveillance of Emily’s internet use |
| --- |

**Explanation:** Exploring the ‘scary’ chat rooms described by Emily encourages her to verbalize her own emotional experiences with the online chat rooms. The word ‘scary’ stands out in Emily’s otherwise blunted responses. Gently exploring that aspect with her and using her own words can facilitate the trust required for Emily (an adolescent) to share more about herself. Tactful questions about Emily’s own cyber-searching suicide would follow the exploration of the ‘scary’ experiences she has already revealed. It would be counterproductive to focus prematurely on Emily’s mother or to lecture Emily on the risks of cyberbullying.

**Potential Challenges for Students:** Again, answers A and B are not incorrect, but are less likely to engage an adolescent during an initial interview.

**Opportunities for Further Discussion:** The facilitator may consider an exploration of the challenges of working with potentially suicidal adolescents and their parents. A discussion about how to maintain confidentiality and trust with an adolescent while meeting the needs/rights of the parents to know would also be a valuable discussion. The internet has become an extensive source of information about suicide, ranging from ‘how to’ tutorials to chat rooms that actually encourage or taunt participants to kill themselves. Data continue to show that adolescents that cyber-search suicide are at increased risk for attempts. Developing strategies to empathically explore this with adolescents is an essential part of the suicide risk assessment.

**Case – Part 3**

The psychiatrist then asks Emily if she has EVER had any suicidal thoughts and she states that when she was 11 years old, she put a plastic bag over her head, but ripped it off when she struggled to breathe. She adds that she NEVER told anyone about it and says that she can’t remember why she did it. She feels embarrassed talking about it, saying “it was a silly thing to do.”

When Emily is asked about her mood, she says she feels guilty that she is not happier. She is napping more and feels tired. Her appetite is increased for “cookies and sweets” and she is afraid she will gain weight. She doesn’t make the effort to see her friends and can’t concentrate. She will miss high school and sighs as she says that she isn’t sure she wants to grow up.

**Question 3**

Before making any treatment recommendations, which of the following is the MOST IMPORTANT issue for the psychiatrist to ask Emily about?

1. **Any history of alcohol or drug use**
2. Any history of sexual abuse
3. Any history of compulsions or obsessions
4. Any history of impulsivity and/or aggression
5. Her relationship with her parents and siblings

**Explanation:** Ignoring or minimizing substance use in adolescents (especially alcohol, opioids and cannabis) will undermine the ability to make an appropriate diagnosis and neutralize the efficacy of any treatment plans. It is essential to assess this issue prior to making any treatment recommendation. Exploring Emily’s relationship with her parents and siblings, including the possibility of present and past abuse and aggression, will remain a focus throughout the evaluation and treatment, but is also influenced by the presence of a substance abuse problem.

**Potential Challenges for Students:** Again, the questions specifically asks for the one question that must be asked before any treatment recommendations can be made. This does not does not mean that asking about her relationship with her parents and siblings (including abuse) is unimportant.

**Opportunities for Further Discussion:** The facilitator may want to include further discussion of ways to identify the presence of substance abuse in adolescents, especially when a teenager denies use. It may also be beneficial to introduce the importance of discussing cannabis use now that it is legal (for adults) in many states.

**Case – Part 4**

Emily and her mother now insist that she must go away to college in September. The psychiatrist recommends that Emily consider deferring her acceptance for a semester, register locally and continue her treatment. The psychiatrist plans to stabilize her mood with a selective serotonin reuptake inhibitor (SSRI), which will be monitored carefully, as well as start psychotherapy to help prepare her for this important next step. Her mother bursts into tears and Emily looks at you and says “I’m going!”

**Question 4**

What is the next best step after empathizing?

1. Reveal Emily’s suicide attempt at age 11 to her mother to emphasize the concern about dire consequences if she goes away to school in September
2. Inform them that studies show that half of college students have suicidal thoughts at some point and that 1 of every 1000 students with ideation will commit suicide
3. **Ask to speak with Emily alone to express the concerns and explore what deferring would mean to her**
4. Ask to speak with Emily’s mother alone to express the concerns and explore why deferring would benefit her

**Explanation:** First and foremost, it is crucial to understand Emily’s perspective on deferring and to demonstrate the willingness to listen to her point of view. Abruptly revealing the prior suicide attempt at age eleven to her mother or requesting the next portion of the interview with her mother would undermine the fledgling therapeutic relationship with Emily. The statistic about suicide among college students is inaccurate. Fortunately, the number of students attending college with suicidal ideation who ultimately commit suicide is lower than for all young adults in that age group.

**Potential Challenges for Students:** There is very little time remaining to construct a plan capable of addressing the concerns for Emily. It is certainly understandable that a psychiatrist would jump to emphasize the concerns for safety in hopes of persuading Emily (and her mother) to delay.

**Opportunities for Further Discussion:** This scenario creates an opportunity to introduce the role of countertransference feelings that emerge when patients reject treatment recommendations and opt for choices that appear dangerous. It also provides an opportunity to discuss obligations and options for a psychiatrist when a patient rejects treatment or is noncompliant.

**Question 5**

| Emily and her mother remain adamant that going away is the best plan for her. Emily denies any current suicidal thoughts but agrees to continue treatment until she goes away. Her mother asks if it would be possible to arrange sessions via a video conference call in the fall. What is the next best step?   1. Recommend weekly sessions until September and then continue with weekly video conference calls while she is away, if she agrees to meet in person during vacations 2. Reinforce the concern and the reasons why it is recommended she postpones her admissions and remain at home in order to have adequate treatment for her depression and cutting 3. Explain that there are concerns and she is not yet stable enough for sessions via a video conference call, therefore she should see a psychiatrist there 4. **Explain to Emily that conducting out of state regular sessions via a video conference call requires that the psychiatrist be licensed to practice medicine in that state** |
| --- |

**Explanation:** Licensing boards currently require that physicians conducting sessions via telepsychiatry obtain additional licensing in the state where the patient resides. Psychiatrists do offer occasional phone sessions when a patient goes temporarily out of state for vacation or work, however, Emily is leaving the state to attend college. Providing regular sessions via telepsychiatry could be viewed as "practicing medicine without a license.” It would be important to explain this to Emily and her mother and then express concern, as well as the need to explore realistic treatment options that will provide adequate safety.

**Potential Challenges for Students**: It is also true that Emily’s current risk of suicide and depression should not be handled long distance, even if it is ‘technically legal’ to do so.

**Opportunities for Further Discussion:** The use of telepsychiatry is growing and a discussion of its risks and benefits (especially if there is a turn to national licensing) may be valuable. For example:

- What are the particular risks of treating suicidal patients this way?
- What are the patient and physician expectations?
- Should the psychiatrist conclude the relationship at the end of the evaluation session or continue to work with Emily until the fall and hope that some better arrangement can be met?

The difficulty in finding psychiatrists (on short notice) who are able to accept new patients due to the shortage of psychiatrists can also be discussed. For example:

- Does it become the psychiatrist’s responsibility to find an out of state psychiatrist?
